# Supplementary material for: Synthesis and Structure of Iron (II) Complexes of Functionalized 1,5-Diaza-3,7-Diphosphacyclooctanes
Source: Molecules. 2020 Aug 19;25(17):3775. doi: 10.3390/molecules25173775 (PMC7503606; doi:10.3390/molecules25173775)

checkCIF/PLATON report

Structure factors have been supplied for datablock(s) shelx

THIS REPORT IS FOR GUIDANCE ONLY. IF USED AS PART OF A REVIEW PROCEDURE FOR PUBLICATION, IT SHOULD NOT REPLACE THE EXPERTISE OF AN EXPERIENCED CRYSTALLOGRAPHIC REFEREE.

No syntax errors found. CIF dictionary Interpreting this report

Datablock: shelx

Bond precision: C-C = 0.0266 A Wavelength=0.71073

Cell:	a=22.651(2)	b=22.651(2)	c=15.3431(17)
	alpha=90	beta=90	gamma=120
Temperature:	100 K		

	Calculated	Reported
Volume	6817.4(14)	6817.3(16)
Space group	P 3 2 1	P 3 2 1
Hall group	P 3 2"	P 3 2"
Moiety formula	3(C68 H80 B F4 Fe N4 P4), 2(B F4), 15(C3 H6 O) [+ solvent]	3(C68 H68 B F4 Fe N4 P4), 2(B F4), 15(C3 H6 O), [+ SOLVENT]
Sum formula	C249 H330 B5 F20 Fe3 N12 O15 P12 [+ solvent]	C249 H294 B5 F20 Fe3 N12 O15 P12
Mr	4704.49	4704.49
Dx,g cm-3	1.146	1.142
Z	1	1
Mu (mm-1)	0.297	0.297
F000	2491.0	2464.0
F000’	2494.33	
h,k,lmax	27,27,18	27,27,18
Nref	8950[4832]	8934
Tmin,Tmax	0.965,0.985	0.468,0.746
Tmin’	0.942	

Correction method= # Reported T Limits: Tmin=0.468 Tmax=0.746
AbsCorr = MULTI-SCAN

Data completeness= 1.85/1.00 Theta(max)= 26.000

R(reflections)= 0.1040(4109) wR2(reflections)= 0.2794(8934)

S = 0.973 Npar= 347

The following ALERTS were generated. Each ALERT has the format
test-name_ALERT_alert-type_alert-level.
Click on the hyperlinks for more details of the test.

Alert level B

```
RINTA01_ALERT_3_B  The value of Rint is greater than 0.18
            Rint given   0.249
PLAT020_ALERT_3_B The Value of Rint is Greater Than 0.12 .........      0.249 Report
PLAT035_ALERT_1_B _chemical_absolute_configuration Info  Not Given     Please Do !
PLAT341_ALERT_3_B Low Bond Precision on  C-C Bonds ...............    0.02664 Ang.
PLAT413_ALERT_2_B Short Inter XH3 .. XHn     H52C     ..H52C     .       1.95 Ang.
                                               2-x,1-x+y,-z  =      6_765 Check
```

Alert level C

```
ABSTY02_ALERT_1_C  An _exptl_absorpt_correction_type has been given without
            a literature citation. This should be contained in the
            _exptl_absorpt_process_details field.
            Absorption correction given as multi-scan
CHEMW01_ALERT_1_C  The difference between the given and expected weight for
            compound is greater 1 mass unit. Check that all hydrogen
            atoms have been taken into account.
PLAT026_ALERT_3_C Ratio Observed / Unique Reflections (too) Low ..        46% Check
PLAT084_ALERT_3_C High wR2 Value (i.e. > 0.25) ...................       0.28 Report
PLAT213_ALERT_2_C Atom C30             has ADP max/min Ratio .....        3.2 prolat
PLAT220_ALERT_2_C NonSolvent   Resd 1  C   Ueq(max)/Ueq(min) Range        3.9 Ratio
PLAT234_ALERT_4_C Large Hirshfeld Difference C33      --C34      .       0.16 Ang.
PLAT241_ALERT_2_C High   'MainMol' Ueq as Compared to Neighbors of        C29 Check
PLAT241_ALERT_2_C High   'MainMol' Ueq as Compared to Neighbors of        C30 Check
PLAT242_ALERT_2_C Low    'MainMol' Ueq as Compared to Neighbors of        C28 Check
PLAT242_ALERT_2_C Low    'MainMol' Ueq as Compared to Neighbors of        C31 Check
PLAT244_ALERT_4_C Low    'Solvent' Ueq as Compared to Neighbors of         B2 Check
PLAT244_ALERT_4_C Low    'Solvent' Ueq as Compared to Neighbors of        C60 Check
PLAT250_ALERT_2_C Large U3/U1 Ratio for Average U(i,j) Tensor ....        2.2 Note
PLAT260_ALERT_2_C Large Average Ueq of Residue Including       O50      0.144 Check
PLAT260_ALERT_2_C Large Average Ueq of Residue Including       O60      0.102 Check
PLAT260_ALERT_2_C Large Average Ueq of Residue Including      O83A      0.164 Check
PLAT331_ALERT_2_C Small Aver Phenyl C-C Dist C11      --C16      .       1.36 Ang.
PLAT905_ALERT_3_C Negative K value in the Analysis of Variance ...     -0.605 Report
PLAT911_ALERT_3_C Missing FCF Refl Between Thmin & STh/L=    0.600          8 Report
```

Alert level G

```
FORMU01_ALERT_2_G  There is a discrepancy between the atom counts in the
            _chemical_formula_sum and the formula from the _atom_site* data.
            Atom count from _chemical_formula_sum:C249 H294 B5 F20 Fe3 N12 O15 P12
            Atom count from the _atom_site data:  C249 H330 B5 F20 Fe3 N12 O15 P12
CELLZ01_ALERT_1_G Difference between formula and atom_site contents detected.
CELLZ01_ALERT_1_G ALERT: Large difference may be due to a
            symmetry error - see SYMMG tests
           From the CIF: _cell_formula_units_Z    1
           From the CIF: _chemical_formula_sum  C249 H294 B5 F20 Fe3 N12 O15 P12
           TEST: Compare cell contents of formula and atom_site data

           atom    Z*formula  cif sites diff
           C        249.00    249.00    0.00
           H        294.00    330.00  -36.00
           B          5.00      5.00    0.00
           F         20.00     20.00    0.00
```

```
          Fe         3.00      3.00    0.00
          N         12.00     12.00    0.00
          O         15.00     15.00    0.00
          P         12.00     12.00    0.00
PLAT002_ALERT_2_G Number of Distance or Angle Restraints on AtSite          8 Note
PLAT041_ALERT_1_G Calc. and Reported SumFormula    Strings  Differ     Please Check
PLAT042_ALERT_1_G Calc. and Reported MoietyFormula Strings  Differ     Please Check
PLAT068_ALERT_1_G Reported F000 Differs from Calcd (or Missing)...     Please Check
PLAT072_ALERT_2_G SHELXL First  Parameter in WGHT  Unusually Large       0.13 Report
PLAT152_ALERT_1_G The Supplied and Calc. Volume s.u. Differ by ...         -2 Units
PLAT171_ALERT_4_G The CIF-Embedded .res File Contains EADP Records         10 Report
PLAT172_ALERT_4_G The CIF-Embedded .res File Contains DFIX Records          4 Report
PLAT173_ALERT_4_G The CIF-Embedded .res File Contains DANG Records          2 Report
PLAT380_ALERT_4_G Incorrectly? Oriented X(sp2)-Methyl Moiety .....        C52 Check
PLAT380_ALERT_4_G Incorrectly? Oriented X(sp2)-Methyl Moiety .....        C82 Check
PLAT606_ALERT_4_G Solvent Accessible VOID(S) in Structure ........          ! Info
PLAT790_ALERT_4_G Centre of Gravity not Within Unit Cell: Resd.  #          3 Note
              C3 H6 O
PLAT790_ALERT_4_G Centre of Gravity not Within Unit Cell: Resd.  #          4 Note
              C3 H6 O
PLAT791_ALERT_4_G Model has Chirality at C9          (Sohnke SpGr)          R Verify
PLAT791_ALERT_4_G Model has Chirality at C24         (Sohnke SpGr)          R Verify
PLAT802_ALERT_4_G CIF Input Record(s) with more than 80 Characters          1 Info
PLAT860_ALERT_3_G Number of Least-Squares Restraints .............          6 Note
PLAT869_ALERT_4_G ALERTS Related to the Use of SQUEEZE Suppressed           ! Info
PLAT883_ALERT_1_G No Info/Value for _atom_sites_solution_primary .     Please Do !
PLAT910_ALERT_3_G Missing # of FCF Reflection(s) Below Theta(Min).          2 Note
PLAT913_ALERT_3_G Missing # of Very Strong Reflections in FCF ....          2 Note
PLAT933_ALERT_2_G Number of OMIT Records in Embedded .res File ...         12 Note
PLAT978_ALERT_2_G Number C-C Bonds with Positive Residual Density.          0 Info
```

```
  0 ALERT level A = Most likely a serious problem - resolve or explain
  5 ALERT level B = A potentially serious problem, consider carefully
 20 ALERT level C = Check. Ensure it is not caused by an omission or oversight
 27 ALERT level G = General information/check it is not something unexpected

 10 ALERT type 1 CIF construction/syntax error, inconsistent or missing data
 17 ALERT type 2 Indicator that the structure model may be wrong or deficient
 10 ALERT type 3 Indicator that the structure quality may be low
 15 ALERT type 4 Improvement, methodology, query or suggestion
  0 ALERT type 5 Informative message, check
```

It is advisable to attempt to resolve as many as possible of the alerts in all categories. Often the minor alerts point to easily fixed oversights, errors and omissions in your CIF or refinement strategy, so attention to these fine details can be worthwhile. In order to resolve some of the more serious problems it may be necessary to carry out additional measurements or structure refinements. However, the purpose of your study may justify the reported deviations and the more serious of these should normally be commented upon in the discussion or experimental section of a paper or in the "special_details" fields of the CIF. checkCIF was carefully designed to identify outliers and unusual parameters, but every test has its limitations and alerts that are not important in a particular case may appear. Conversely, the absence of alerts does not guarantee there are no aspects of the results needing attention. It is up to the individual to critically assess their own results and, if necessary, seek expert advice.

Publication of your CIF in IUCr journals

A basic structural check has been run on your CIF. These basic checks will be run on all CIFs submitted for publication in IUCr journals (*Acta Crystallographica*, *Journal of Applied Crystallography*, *Journal of Synchrotron Radiation*); however, if you intend to submit to *Acta Crystallographica Section C* or *E* or *IUCrData*, you should make sure that full publication checks are run on the final version of your CIF prior to submission.

Publication of your CIF in other journals

Please refer to the *Notes for Authors* of the relevant journal for any special instructions relating to CIF submission.

PLATON version of 16/07/2020; check.def file version of 12/07/2020

Datablock shelx - ellipsoid plot

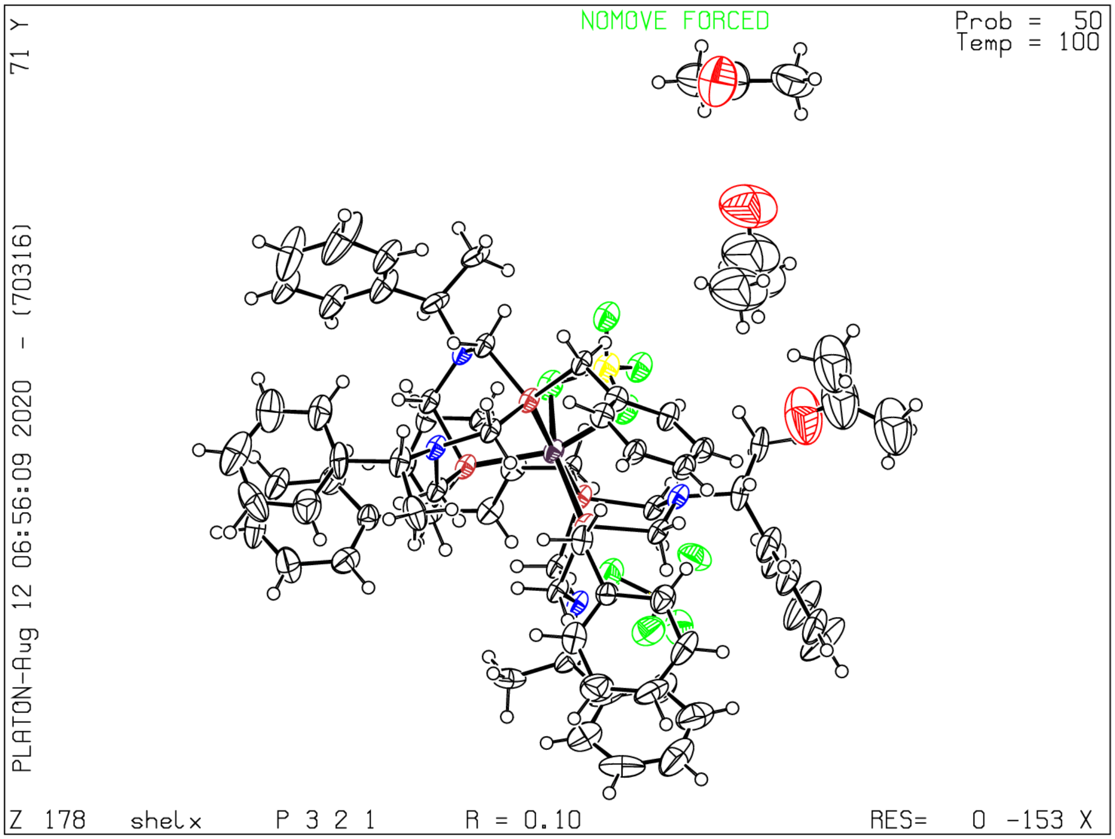

Supplement: Supplementary file 1 [file molecules-25-03775-s001.zip › molecules-893777-final-supplementary/checkcif-11b-resub.pdf]
