# Supplementary material for: Synthesis and Structure of Iron (II) Complexes of Functionalized 1,5-Diaza-3,7-Diphosphacyclooctanes
Source: Molecules. 2020 Aug 19;25(17):3775. doi: 10.3390/molecules25173775 (PMC7503606; doi:10.3390/molecules25173775)

# checkCIF/PLATON report

Structure factors have been supplied for datablock(s) I

THIS REPORT IS FOR GUIDANCE ONLY. IF USED AS PART OF A REVIEW PROCEDURE FOR PUBLICATION, IT SHOULD NOT REPLACE THE EXPERTISE OF AN EXPERIENCED CRYSTALLOGRAPHIC REFEREE.

No syntax errors found. CIF dictionary Interpreting this report

## Datablock: I

---

Bond precision: C-C = 0.0232 A Wavelength=0.71073

| | | | |
|---|---|---|---|
| Cell: | a=13.7178(14) | b=20.452(2) | c=27.659(3) |
| | alpha=90 | beta=90 | gamma=90 |
| Temperature: | 100 K | | |

| | Calculated | Reported |
|---|---|---|
| Volume | 7759.9(14) | 7759.8(14) |
| Space group | P 21 21 21 | P 21 21 21 |
| Hall group | P 2ac 2ab | P 2ac 2ab |
| Moiety formula | C72 H86 Fe N6 P4, 2(B F4), 2(C2 H3 N) | C72 H86 Fe N6 P4, 2(B F4), 2(C2 H3 N) |
| Sum formula | C76 H92 B2 F8 Fe N8 P4 | C76 H92 B2 F8 Fe N8 P4 |
| Mr | 1470.93 | 1470.92 |
| Dx,g cm-3 | 1.259 | 1.259 |
| Z | 4 | 4 |
| Mu (mm-1) | 0.342 | 0.342 |
| F000 | 3088.0 | 3088.0 |
| F000' | 3092.27 | |
| h,k,lmax | 16,25,33 | 16,24,33 |
| Nref | 14986[ 8195] | 14690 |
| Tmin,Tmax | 0.960,0.983 | 0.619,0.746 |
| Tmin' | 0.934 | |

Correction method= # Reported T Limits: Tmin=0.619 Tmax=0.746
AbsCorr = MULTI-SCAN

Data completeness= 1.79/0.98 Theta(max)= 25.842

R(reflections)= 0.0791( 7289) wR2(reflections)= 0.2081( 14690)

S = 1.007 Npar= 866

---

The following ALERTS were generated. Each ALERT has the format
**test-name_ALERT_alert-type_alert-level**.
Click on the hyperlinks for more details of the test.

---

## Alert level B

```
RINTA01_ALERT_3_B  The value of Rint is greater than 0.18
            Rint given   0.197
PLAT020_ALERT_3_B The Value of Rint is Greater Than 0.12 .........      0.197 Report
PLAT341_ALERT_3_B Low Bond Precision on  C-C Bonds ...............    0.02317 Ang.
PLAT910_ALERT_3_B Missing # of FCF Reflection(s) Below Theta(Min).         15 Note
```

---

## Alert level C

```
ABSTY02_ALERT_1_C  An _exptl_absorpt_correction_type has been given without
            a literature citation. This should be contained in the
            _exptl_absorpt_process_details field.
            Absorption correction given as multi-scan
PLAT213_ALERT_2_C Atom C13             has ADP max/min Ratio .....        3.1 prolat
PLAT220_ALERT_2_C NonSolvent Resd 1  C   Ueq(max) / Ueq(min) Range        4.6 Ratio
PLAT222_ALERT_3_C NonSolvent Resd 1  H   Uiso(max)/Uiso(min) Range        4.6 Ratio
PLAT234_ALERT_4_C Large Hirshfeld Difference C33B     --C34B     .       0.18 Ang.
PLAT234_ALERT_4_C Large Hirshfeld Difference C36      --C37      .       0.19 Ang.
PLAT241_ALERT_2_C High   'MainMol' Ueq as Compared to Neighbors of        C13 Check
PLAT241_ALERT_2_C High   'MainMol' Ueq as Compared to Neighbors of       C13B Check
PLAT241_ALERT_2_C High   'MainMol' Ueq as Compared to Neighbors of       C26B Check
PLAT241_ALERT_2_C High   'MainMol' Ueq as Compared to Neighbors of        C29 Check
PLAT241_ALERT_2_C High   'MainMol' Ueq as Compared to Neighbors of       C36B Check
PLAT242_ALERT_2_C Low    'MainMol' Ueq as Compared to Neighbors of       C18B Check
PLAT242_ALERT_2_C Low    'MainMol' Ueq as Compared to Neighbors of       C24B Check
PLAT242_ALERT_2_C Low    'MainMol' Ueq as Compared to Neighbors of       C33B Check
PLAT242_ALERT_2_C Low    'MainMol' Ueq as Compared to Neighbors of        C41 Check
PLAT244_ALERT_4_C Low    'Solvent' Ueq as Compared to Neighbors of        C51 Check
PLAT244_ALERT_4_C Low    'Solvent' Ueq as Compared to Neighbors of        C61 Check
PLAT260_ALERT_2_C Large Average Ueq of Residue Including        F1      0.211 Check
PLAT260_ALERT_2_C Large Average Ueq of Residue Including        F5      0.265 Check
PLAT260_ALERT_2_C Large Average Ueq of Residue Including       N50      0.162 Check
PLAT260_ALERT_2_C Large Average Ueq of Residue Including       N60      0.182 Check
PLAT332_ALERT_2_C Large Phenyl C-C Range     C26B      -C31B     .       0.20 Ang.
PLAT601_ALERT_2_C Structure Contains Solvent Accessible VOIDS of .         37 Ang**3
PLAT761_ALERT_1_C CIF Contains no X-H Bonds ......................     Please Check
PLAT762_ALERT_1_C CIF Contains no X-Y-H or H-Y-H Angles ..........     Please Check
PLAT911_ALERT_3_C Missing FCF Refl Between Thmin & STh/L=    0.600          2 Report
```

---

## Alert level G

```
PLAT002_ALERT_2_G Number of Distance or Angle Restraints on AtSite          5 Note
PLAT083_ALERT_2_G SHELXL Second Parameter in WGHT  Unusually Large      10.32 Why ?
PLAT171_ALERT_4_G The CIF-Embedded .res File Contains EADP Records          1 Report
PLAT172_ALERT_4_G The CIF-Embedded .res File Contains DFIX Records          4 Report
PLAT244_ALERT_4_G Low    'Solvent' Ueq as Compared to Neighbors of         B1 Check
PLAT244_ALERT_4_G Low    'Solvent' Ueq as Compared to Neighbors of         B2 Check
PLAT720_ALERT_4_G Number of Unusual/Non-Standard Labels ..........          8 Note
PLAT790_ALERT_4_G Centre of Gravity not Within Unit Cell: Resd.  #          5 Note
              C2 H3 N
PLAT791_ALERT_4_G Model has Chirality at P3B        (Sohnke SpGr)           R Verify
PLAT791_ALERT_4_G Model has Chirality at P7B        (Sohnke SpGr)           S Verify
PLAT791_ALERT_4_G Model has Chirality at C9         (Sohnke SpGr)           R Verify
PLAT791_ALERT_4_G Model has Chirality at C9B        (Sohnke SpGr)           R Verify
PLAT791_ALERT_4_G Model has Chirality at C24        (Sohnke SpGr)           R Verify
PLAT791_ALERT_4_G Model has Chirality at C24B       (Sohnke SpGr)           R Verify
```

```
PLAT802_ALERT_4_G CIF Input Record(s) with more than 80 Characters          1 Info
PLAT860_ALERT_3_G Number of Least-Squares Restraints .............          4 Note
PLAT883_ALERT_1_G No Info/Value for _atom_sites_solution_primary .     Please Do !
PLAT912_ALERT_4_G Missing # of FCF Reflections Above STh/L=  0.600         92 Note
PLAT933_ALERT_2_G Number of OMIT Records in Embedded .res File ...         17 Note
PLAT978_ALERT_2_G Number C-C Bonds with Positive Residual Density.          0 Info
```

---

0 **ALERT level A** = Most likely a serious problem - resolve or explain
4 **ALERT level B** = A potentially serious problem, consider carefully
26 **ALERT level C** = Check. Ensure it is not caused by an omission or oversight
20 **ALERT level G** = General information/check it is not something unexpected

4 ALERT type 1 CIF construction/syntax error, inconsistent or missing data
21 ALERT type 2 Indicator that the structure model may be wrong or deficient
7 ALERT type 3 Indicator that the structure quality may be low
18 ALERT type 4 Improvement, methodology, query or suggestion
0 ALERT type 5 Informative message, check

---

# checkCIF publication errors

---

## Alert level A

```
PUBL004_ALERT_1_A  The contact author's name and address are missing,
            _publ_contact_author_name and _publ_contact_author_address.
PUBL005_ALERT_1_A  _publ_contact_author_email, _publ_contact_author_fax and
            _publ_contact_author_phone are all missing.
            At least one of these should be present.
PUBL006_ALERT_1_A  _publ_requested_journal is missing
            e.g. 'Acta Crystallographica Section C'
PUBL008_ALERT_1_A  _publ_section_title is missing. Title of paper.
PUBL012_ALERT_1_A  _publ_section_abstract is missing.
            Abstract of paper in English.
```

---

## Alert level G

```
PUBL017_ALERT_1_G The _publ_section_references section is missing or
            empty.
```

---

5 **ALERT level A** = Data missing that is essential or data in wrong format
1 **ALERT level G** = General alerts. Data that may be required is missing

---

**Publication of your CIF**

You should attempt to resolve as many as possible of the alerts in all categories. Often the minor alerts point to easily fixed oversights, errors and omissions in your CIF or refinement strategy, so attention to these fine details can be worthwhile. In order to resolve some of the more serious problems it may be necessary to carry out additional measurements or structure refinements. However, the nature of your study may justify the reported deviations from journal submission requirements and the more serious of these should be commented upon in the discussion or experimental section of a paper or in the "special_details" fields of the CIF. *checkCIF* was carefully designed to identify outliers and unusual parameters, but every test has its limitations and alerts that are not important in a particular case may appear. Conversely, the absence of alerts does not guarantee there are no aspects of the results needing attention. It is up to the individual to critically assess their own results and, if necessary, seek expert advice.

If level A alerts remain, which you believe to be justified deviations, and you intend to submit this CIF for publication in a journal, you should additionally insert an explanation in your CIF using the Validation Reply Form (VRF) below. This will allow your explanation to be considered as part of the review process.

**Validation response form**

Please find below a validation response form (VRF) that can be filled in and pasted into your CIF.

```
# start Validation Reply Form
_vrf_PUBL004_GLOBAL
;
PROBLEM: The contact author's name and address are missing,
RESPONSE: ...
;
_vrf_PUBL005_GLOBAL
;
PROBLEM: _publ_contact_author_email, _publ_contact_author_fax and
RESPONSE: ...
;
_vrf_PUBL006_GLOBAL
;
PROBLEM: _publ_requested_journal is missing
RESPONSE: ...
;
_vrf_PUBL008_GLOBAL
;
PROBLEM: _publ_section_title is missing. Title of paper.
RESPONSE: ...
;
_vrf_PUBL012_GLOBAL
;
PROBLEM: _publ_section_abstract is missing.
RESPONSE: ...
;
# end Validation Reply Form
```

If you wish to submit your CIF for publication in Acta Crystallographica Section C or E, you should upload your CIF via the web. If you wish to submit your CIF for publication in IUCrData you should upload your CIF via the web. If your CIF is to form part of a submission to another IUCr journal, you will be asked, either during electronic submission or by the Co-editor handling your paper, to upload your CIF via our web site.

---

## PLATON version of 22/04/2020; check.def file version of 09/03/2020

**Datablock I** - ellipsoid plot

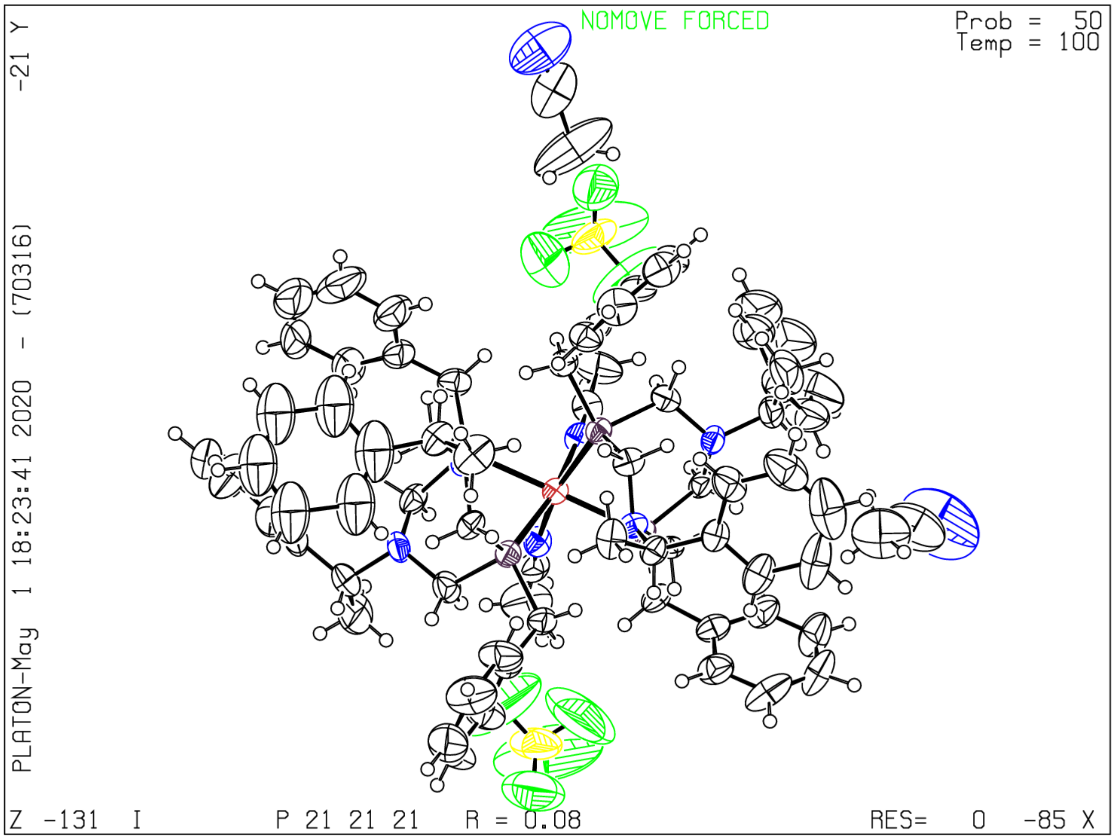

Supplement: Supplementary file 1 [file molecules-25-03775-s001.zip › molecules-893777-final-supplementary/checkcif-8.pdf]
