# Supplementary material for: Synthesis and Structure of Iron (II) Complexes of Functionalized 1,5-Diaza-3,7-Diphosphacyclooctanes
Source: Molecules. 2020 Aug 19;25(17):3775. doi: 10.3390/molecules25173775 (PMC7503606; doi:10.3390/molecules25173775)
Supplement: Supplementary file 1 [file molecules-25-03775-s001.zip › molecules-893777-final-supplementary/ESI-spectra.pdf]

Electronic Supporting Information

Synthesis and structure of iron (II) complexes of functionalized 1,5-diaza-3,7-diphosphacyclooctanes.

Yulia S. Spiridonova, Yulia A. Nikolaeva, Anna S. Balueva*, Elvira I. Musina, Igor A. Litvinov, Igor D. Strelnik, Vera V. Khrizanforova, Yulia G. Budnikova, Andrey A. Karasik

Arbuzov Institute of Organic and Physical Chemistry, FRC Kazan Scientific Center, Russian Academy of Sciences, Arbuzov str. 8, 420088, Kazan, Russian Federation; arbuzov@iopc.ru

* Correspondence: balueva62@mai.ru; Tel.: (+7843)2727392

Content

General methods	2
The spectra of the synthesized compounds	2
The spectra of the ligand **3**	2
The spectra of the ligand **5**	3
The spectra of the complex **6**	4
The spectra of the complex **7**	6
The spectra of the complex **8**	8
The spectra of the complex **9**	10
The spectra of the complex **10**	12
The spectra and TG/DSC diagram of the complex **11b**	14
The spectra of the complex **12**	17
The spectra of the complex **13**	19

General methods: ESI_{pos} MS were recorded with an AmazonX (Bruker Daltonic GmbH, Bremen, Germany) spectrometer at a capillary voltage of 4500 V. DataAnalysis 4.0 (Bruker Daltonic GmbH, Bremen, Germany) program was used to process the mass spectrometry data. MALDI MS were recorded with an Ultraflex III TOF/TOF (Bruker Daltonics, Germany) spectrometer on p-nitroaniline matrix. FlexAnalysis 3.0 (Bruker Daltonics) program was used to process the mass spectrometry data. The mass spectra are reported as m/z values. ^{1}H NMR (400 MHz and 600 MHz) and ^{31}P NMR (162 and 242 MHz) spectra were obtained with Bruker Avance-DRX 400 and Bruker Avance-600 spectrometers. The chemical shifts are reported in ppm relative to $SiMe_4$ (^{1}H, internal standard), and 85% H_3PO_4 (aq) (^{31}P; external standard). The coupling constants (J) are reported in Hz. Simultaneous thermogravimetry and differential scanning calorimetry (TG/DSC) analysis of samples of **11b** (solvate) (10.7 mg) were performed using the STA 449F1 Jupiter (Netzsch, Germany) thermoanalyzer (in the range of temperatures from 40 to 200 °C in aluminum crucible under dynamic atmosphere of argon (75 ml/min). The heating rate was 10 °C/min.

The spectra of the synthesized compounds.

3,7-dibenzyl-1,5-di(1'-(*R*)-phenylethyl)-1,5-diaza-3,7-diphosphacyclooctane (3).

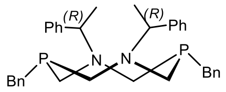

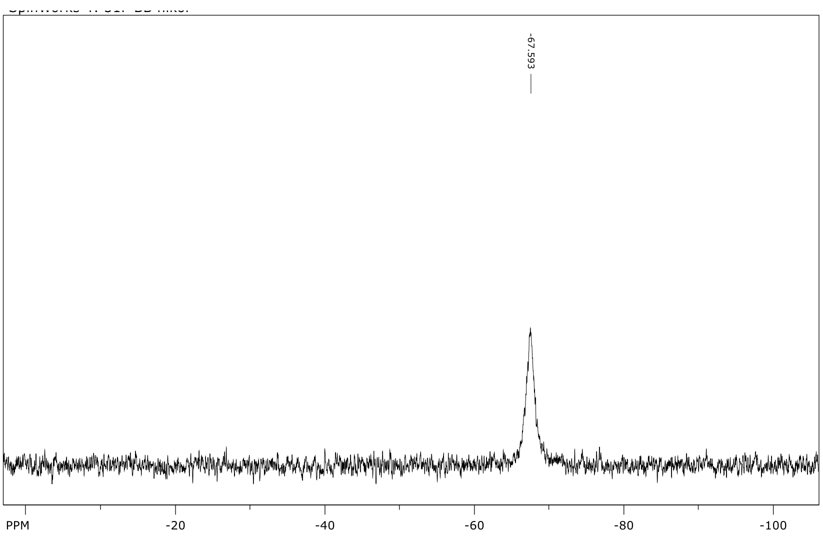

Figure S1. ^{31}P NMR spectrum of **3** in $CDCl_3$ (162 MHz).

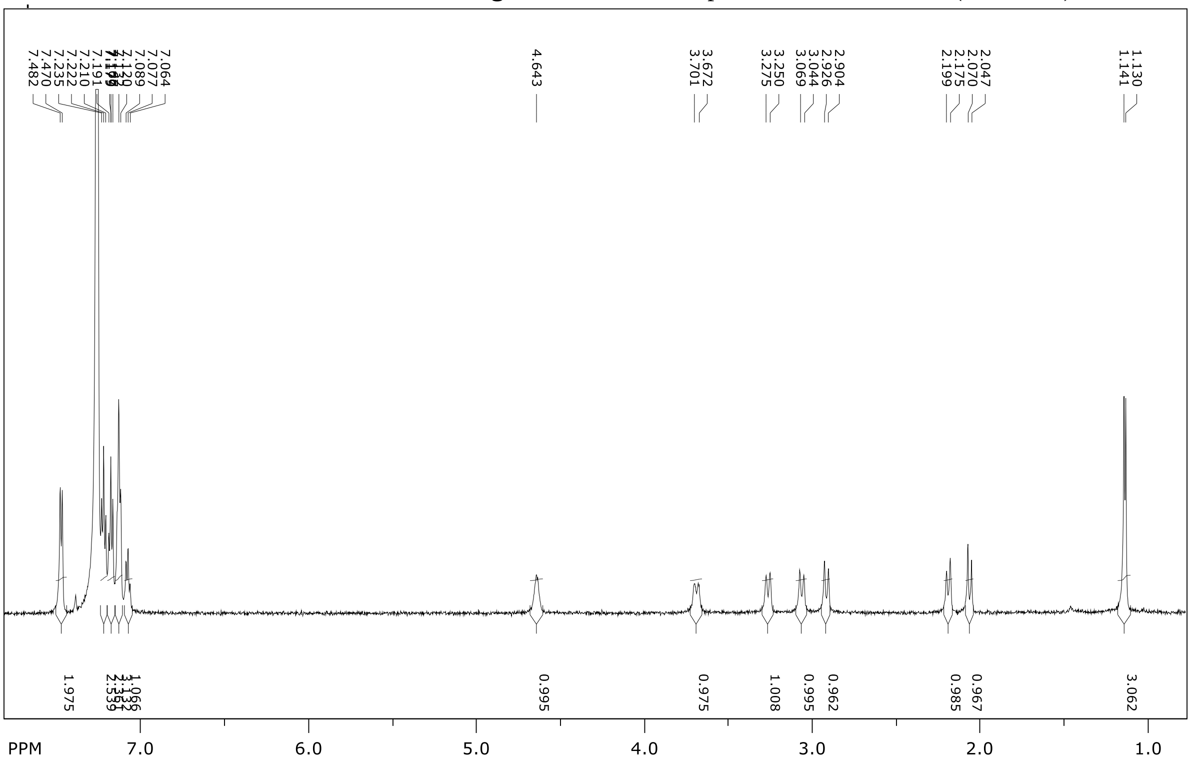

Figure S2. ^{1}H NMR spectrum of **3** ($CDCl_3$,400 MHz).

3,7-dimesityl-1,5-di(p-tolyl)-1,5-diaza-3,7-diphosphacyclooctane (5)

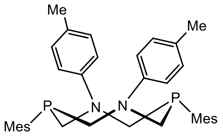

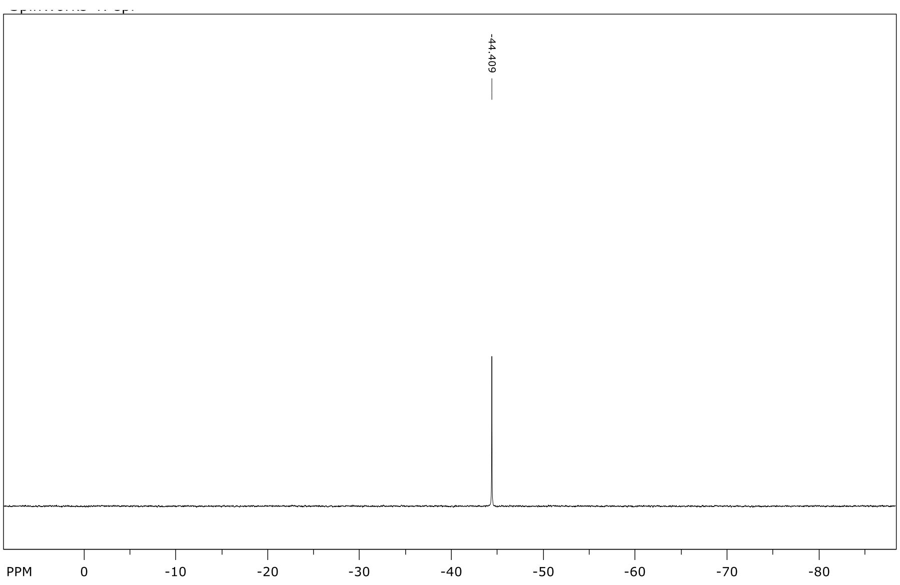

Figure S3. ^{31}P NMR spectrum of **5** ($CDCl_3$,162 MHz).

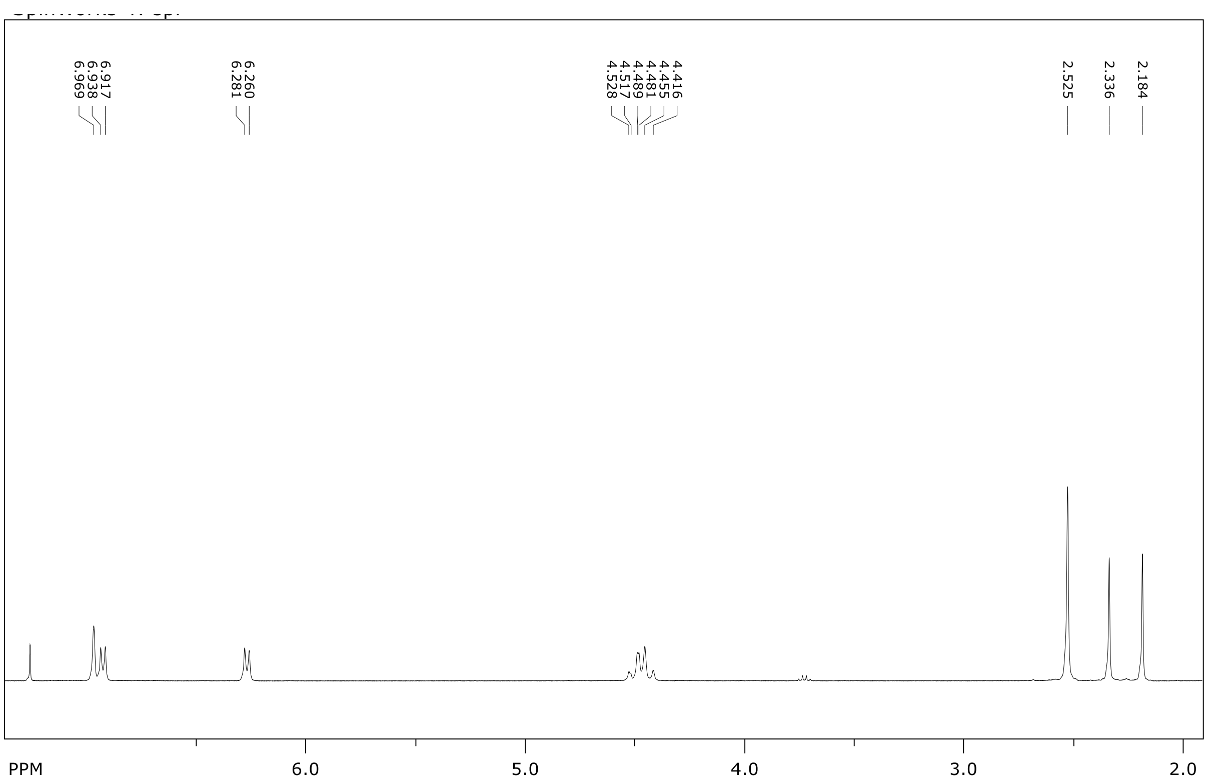

Figure S4. ^{1}H NMR spectrum of **5** ($CDCl_3$, 400 MHz).

Bis(3,7-diphenyl-1,5-di(*p*-methoxyphenyl)-1,5-diaza-3,7-diphosphacyclooctane)-bis(acetonitrile)iron(II)] tetrafluoroborate (6)

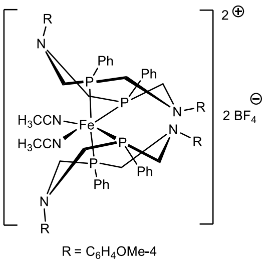

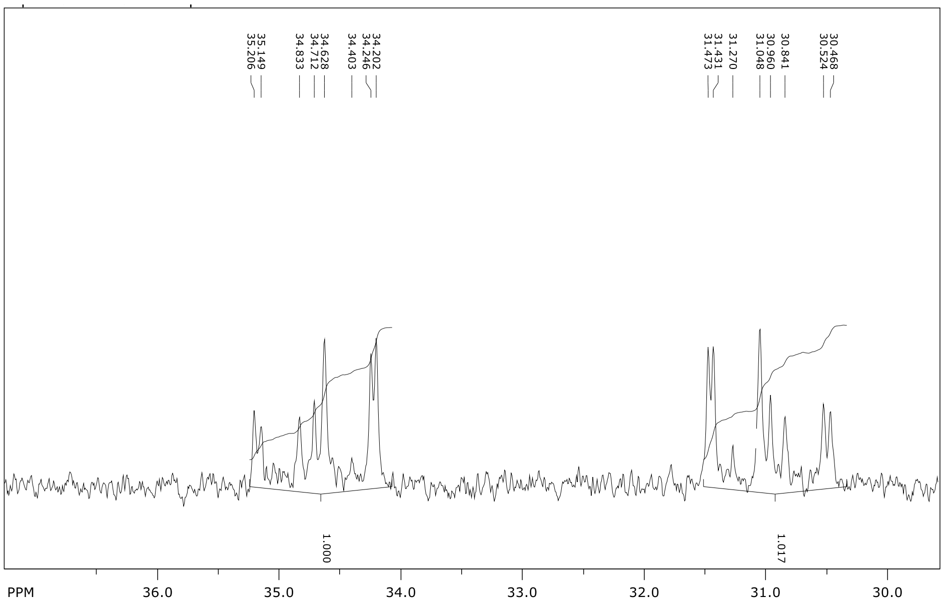

Figure S5. ^{31}P NMR spectrum of **6** (CD_3CN, 162 MHz).

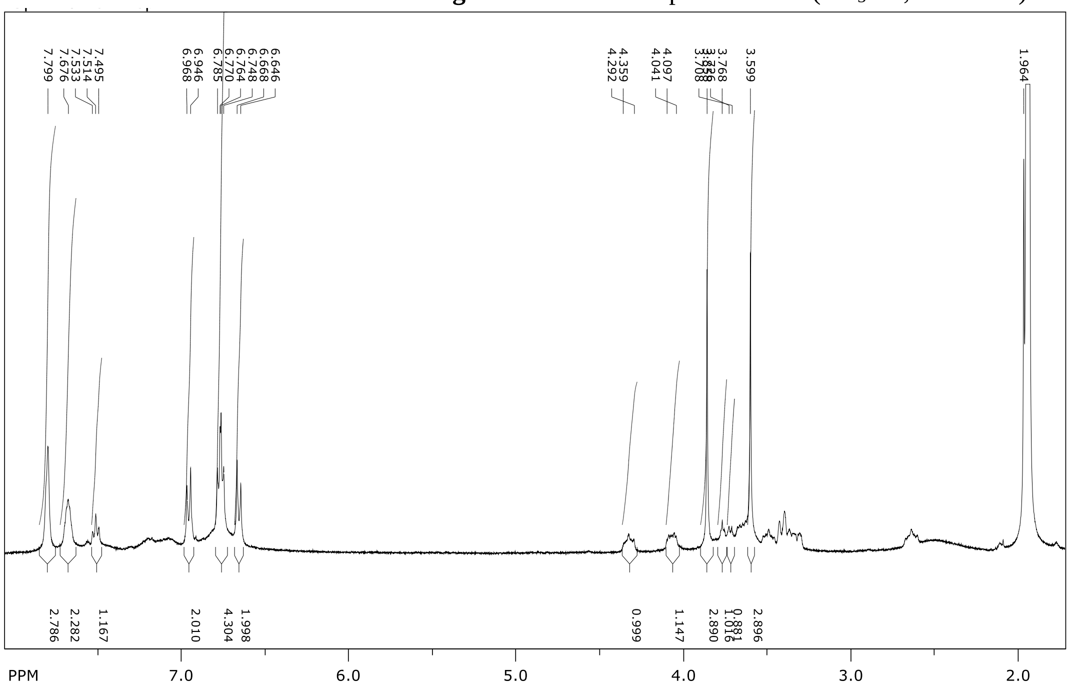

Figure S6. ^1H NMR spectrum of **6** (CD_3CN, 400 MHz).

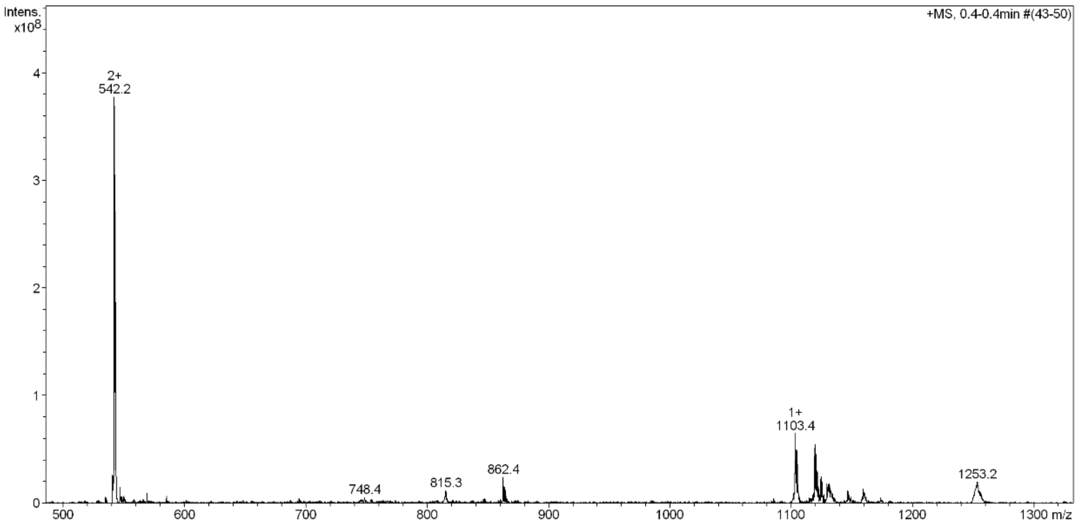

Figure S7. ESI-MS spectrum of **6** (CD_3CN).

[Bis(3,7-dibenzyl-1,5-diphenyl-1,5-diaza-3,7-diphosphacyclooctane)-bis(acetonitrile)iron(II)] tetrafluoroborate (7).

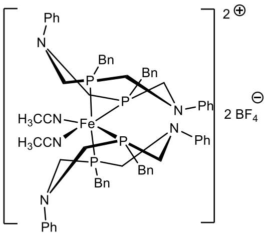

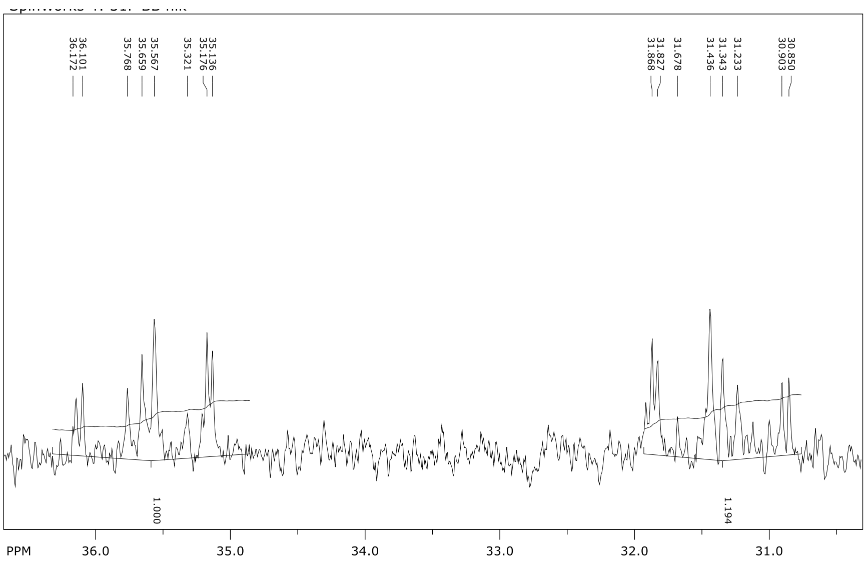

Figure S8. ^{31}P NMR spectrum of **7** (CD_3CN, 162 MHz).

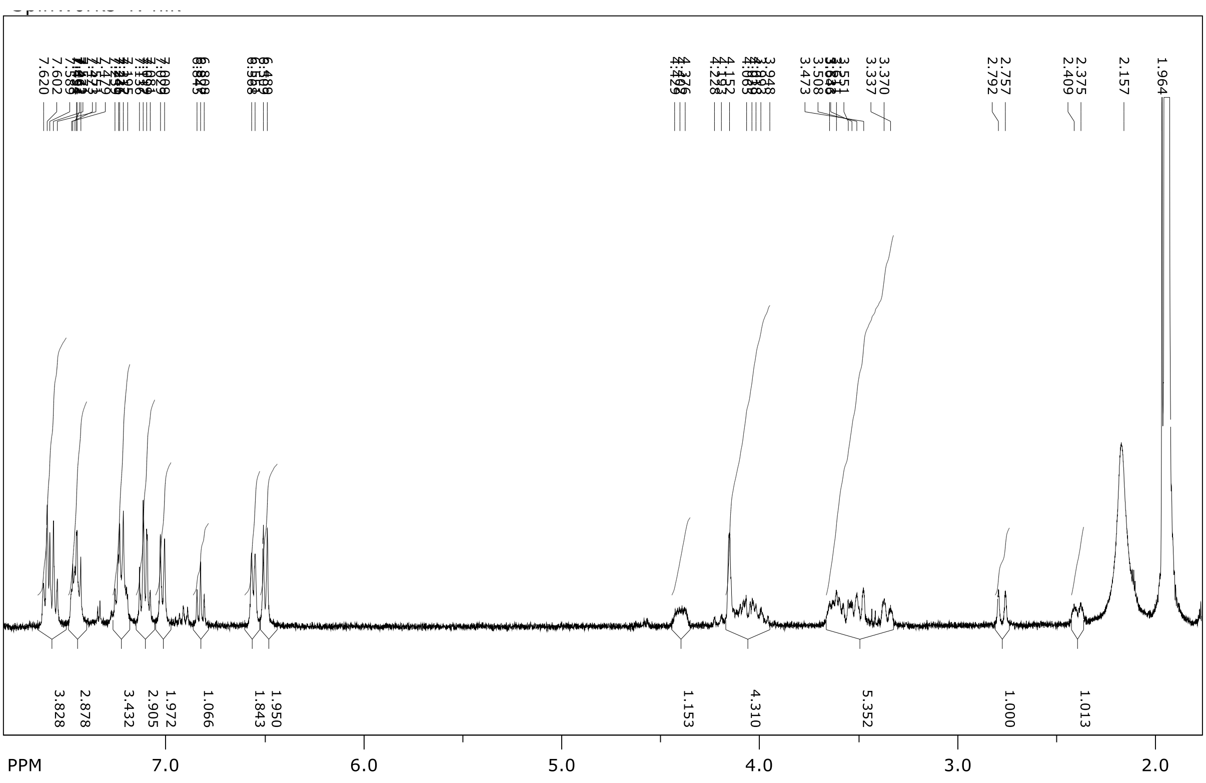

Figure S9. ^1H NMR spectrum of **7** (CD_3CN, 400 MHz) (2.16 s – H_2O in the solvent).

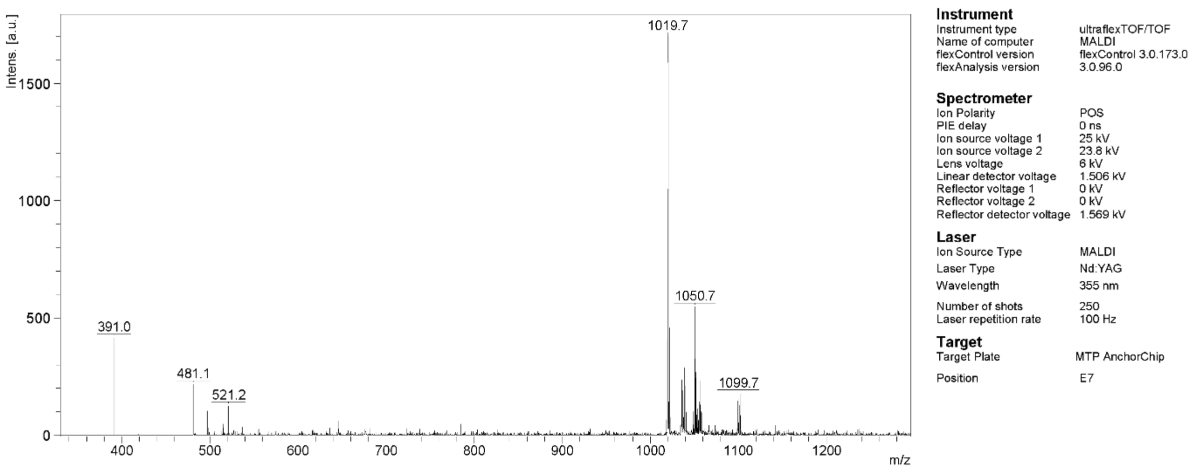

Figure S10. MALDI-MS spectrum of **7** (CD_3CN).

[Bis(3,7-dibenzyl-1,5-di(1’-(*R*)-phenylethyl)-1,5-diaza-3,7-diphosphacyclooctane)-bis(acetonitrile)iron(II)] tetrafluoroborate (8).

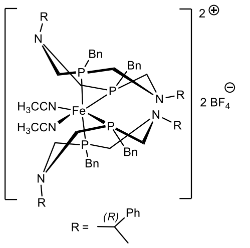

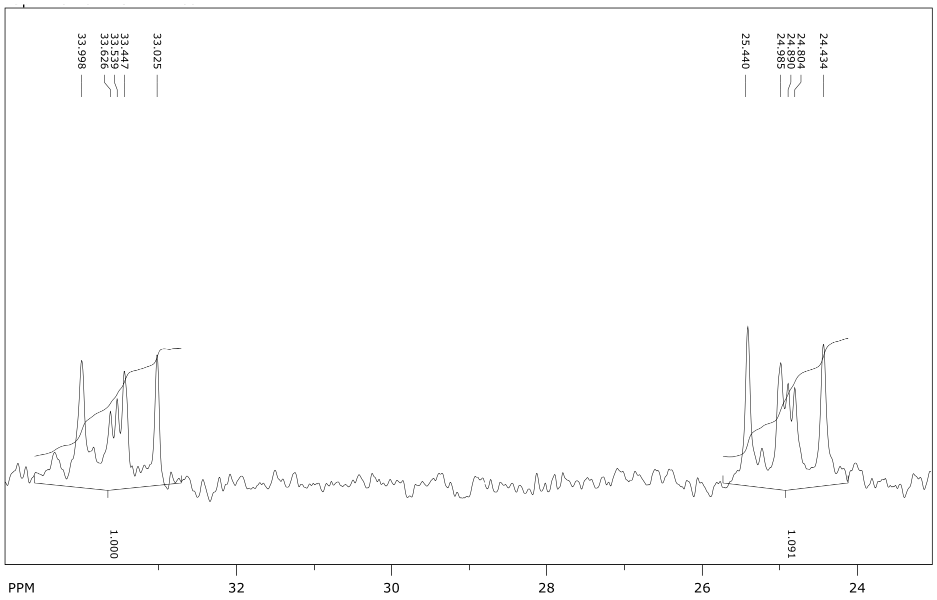

Figure S11. ^{31}P NMR spectrum of **8** (CD_3CN, 162 MHz).

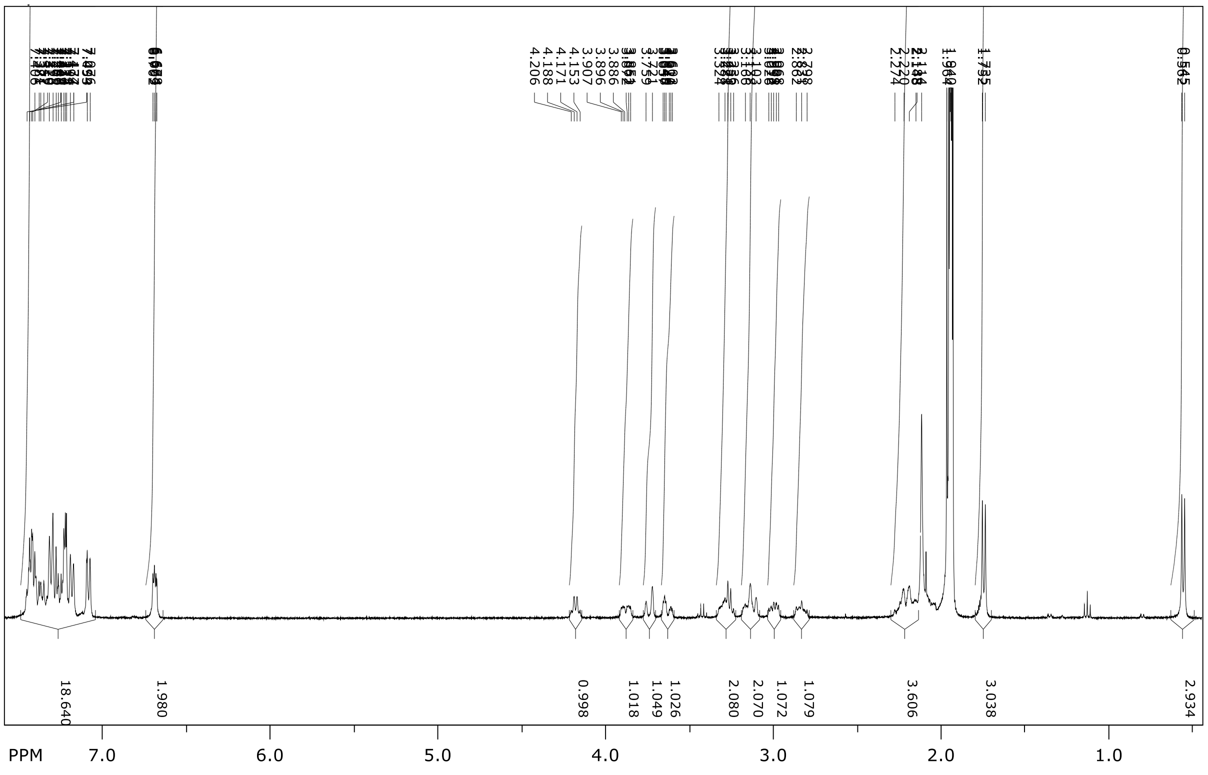

Figure S12. ^{1}H NMR spectrum of **8** (CD_3CN, 400 MHz) (2.11 s – H_2O in the solvent).

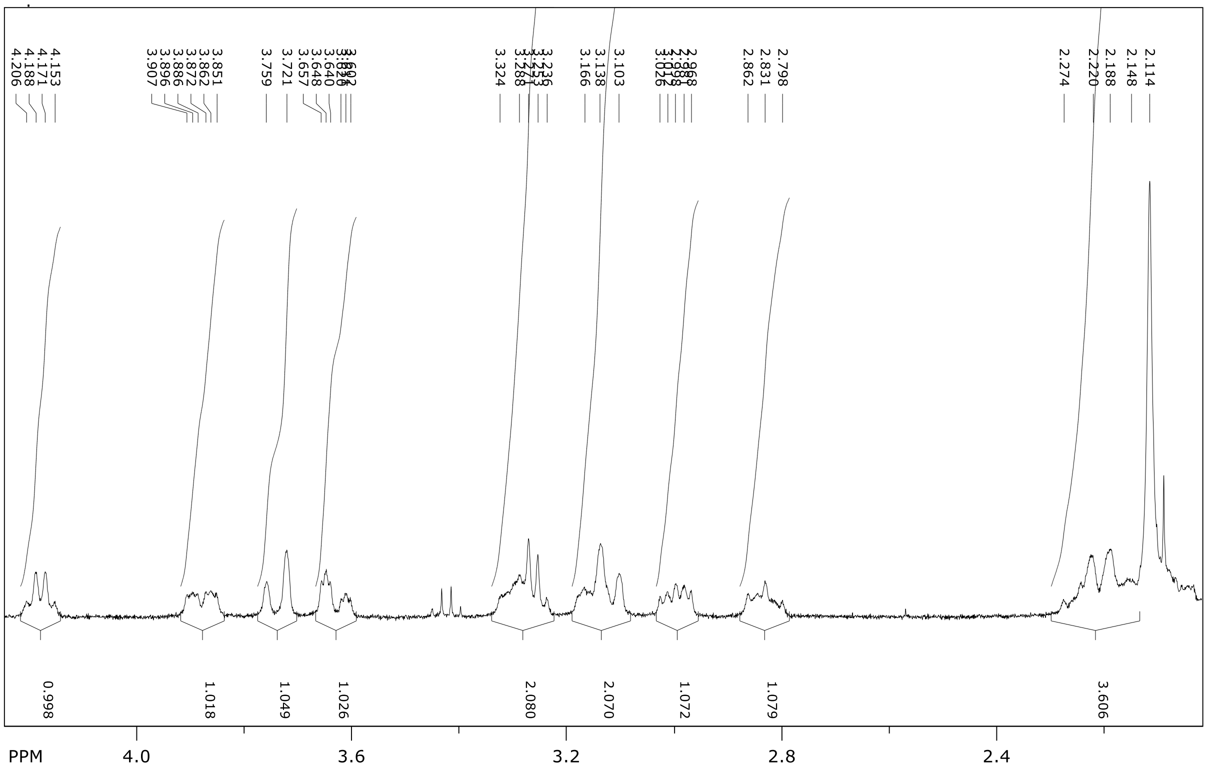

Figure S13. ^{1}H NMR spectrum of **8** (CD_3CN, 400 MHz), the region of protons of P-CH_2-N, P-CH_2-Ph, CH(Ph)Me fragments (2.11 s – H_2O in the solvent).

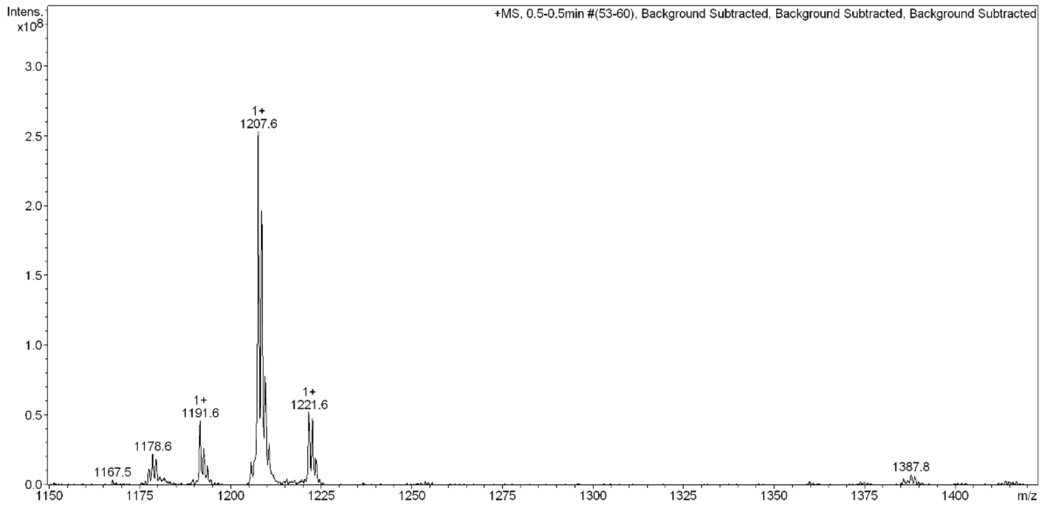

Figure S14. ESI-MS spectrum of **8** (CD_3CN).

[Bis(3,7-di(pyridine-2’-yl)-1,5-di(p-tolyl)-1,5-diaza-3,7-diphosphacyclooctane)-bis(acetonitrile)iron(II)] tetrafluoroborate (9).

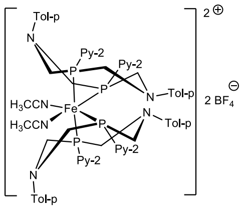

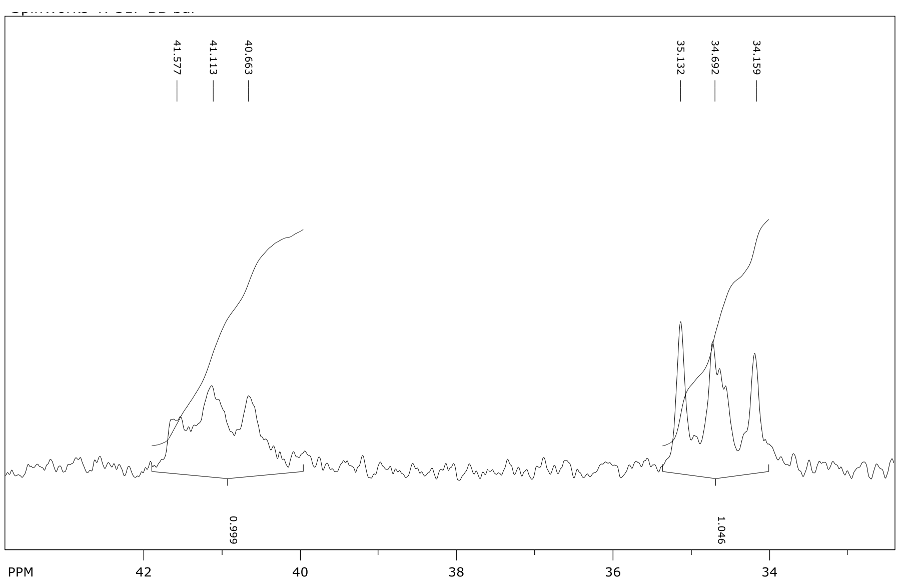

Figure S15. ^{31}P NMR spectrum of **9** (CD_3CN, 162 MHz).

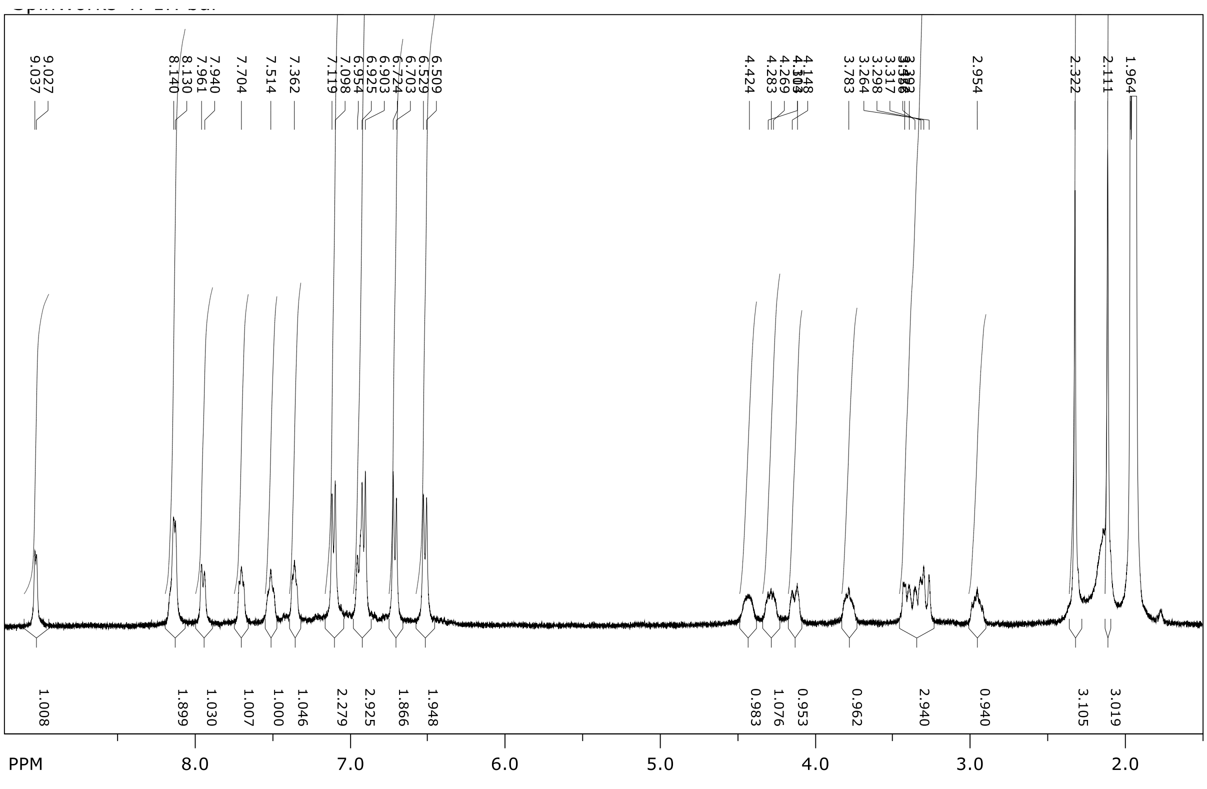

Figure S16. ^{1}H NMR spectrum of **9** (CD_3CN, 400 MHz).

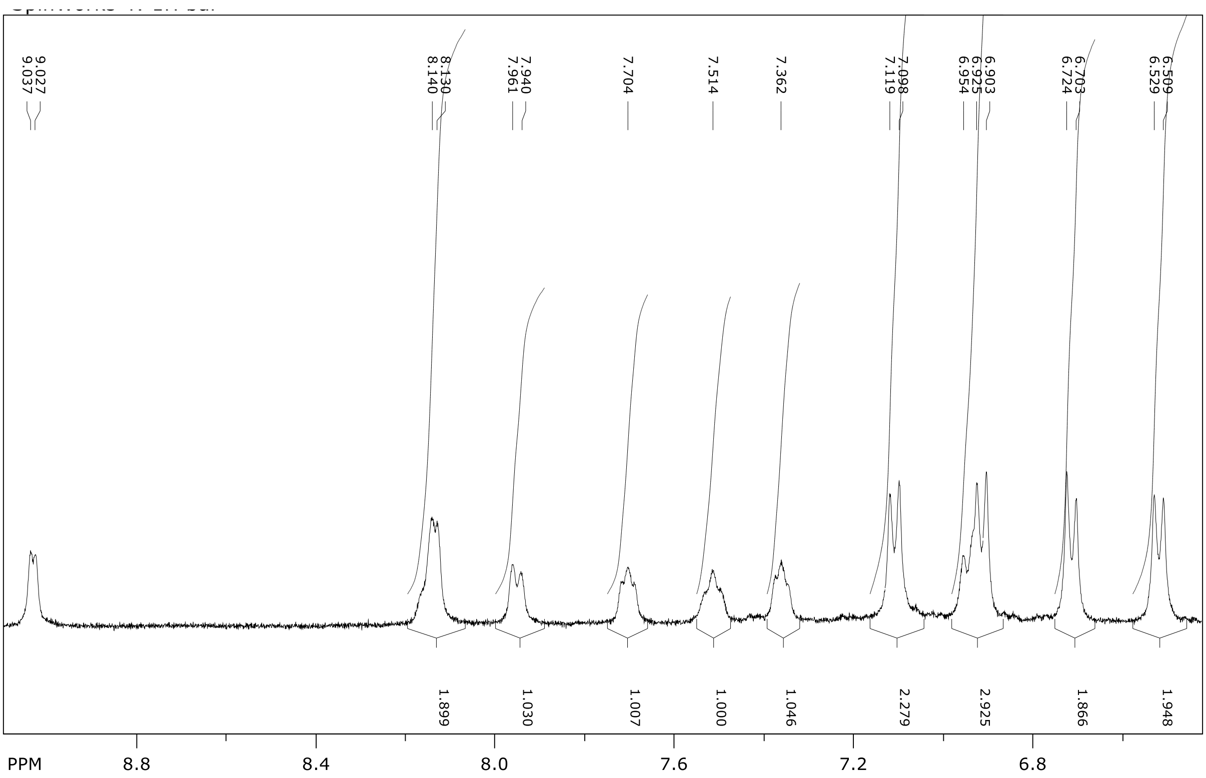

Figure S17. ^{1}H NMR spectrum of **9**, the region of aromatic protons (CD_3CN, 400 MHz).

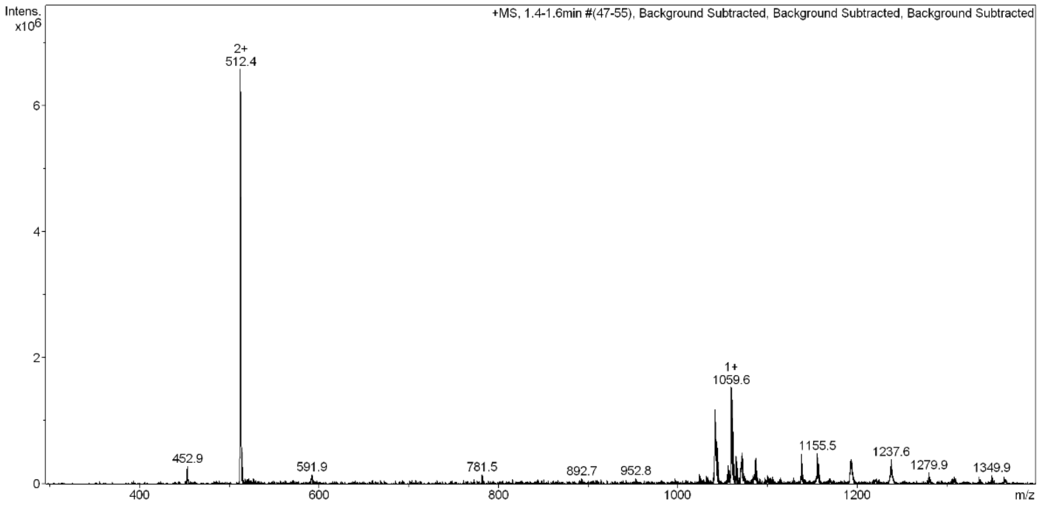

Figure S18. ESI-MS spectrum of **9** (CD_3CN).

[(3,7-dimesityl-1,5-di(p-tolyl)-1,5-diaza-3,7-diphosphacyclooctane)tetrakis-(acetonitrile)iron(II)] tetrafluoroborate (10).

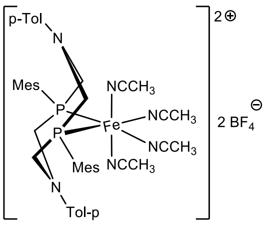

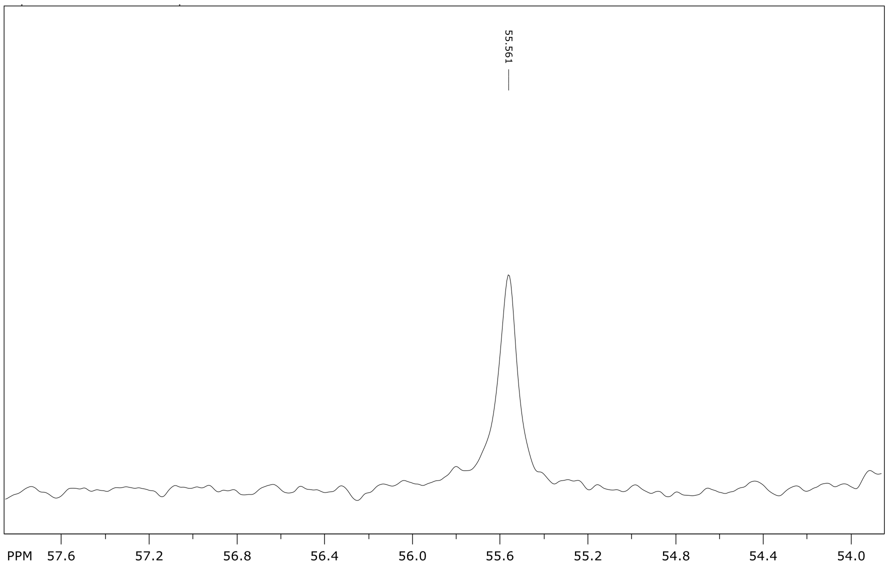

Figure S19. ^{31}P NMR spectrum of **10** (CD_3CN, 162 MHz).

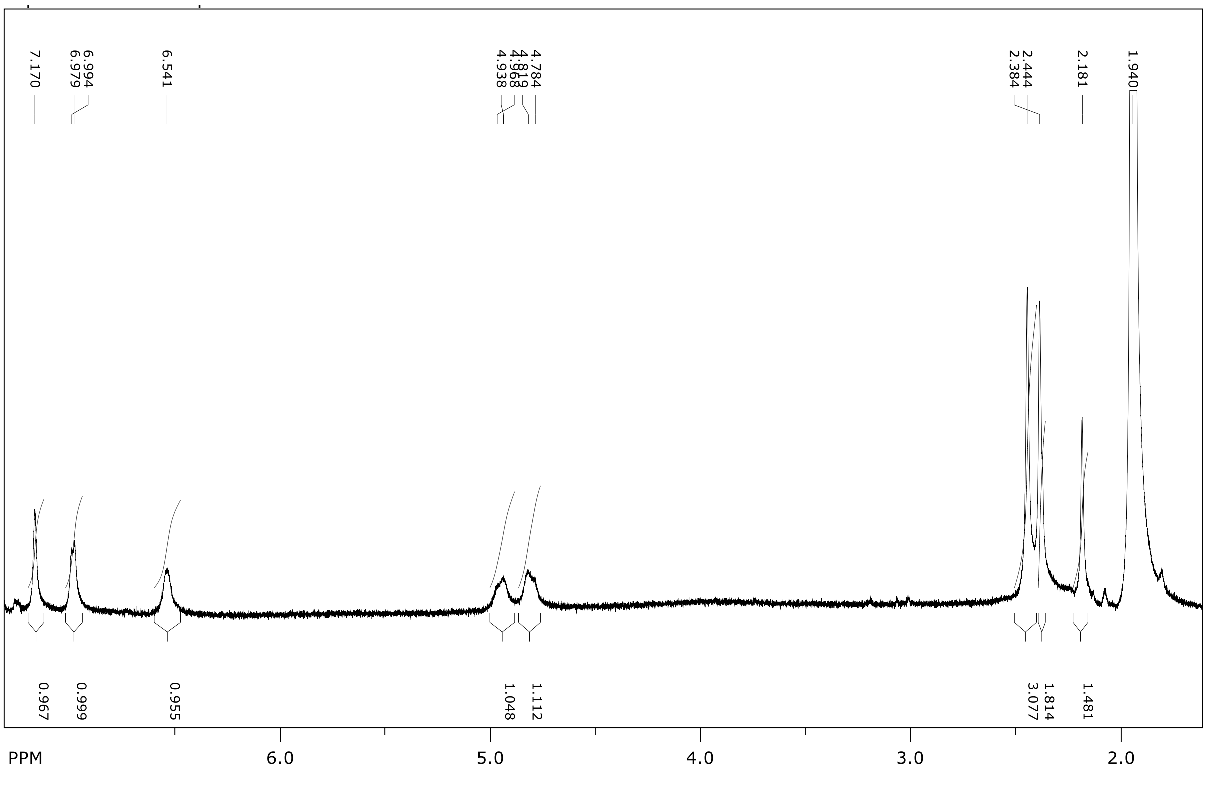

Figure S20. ^{1}H NMR spectrum of **10** (CD_3CN, 400 MHz).

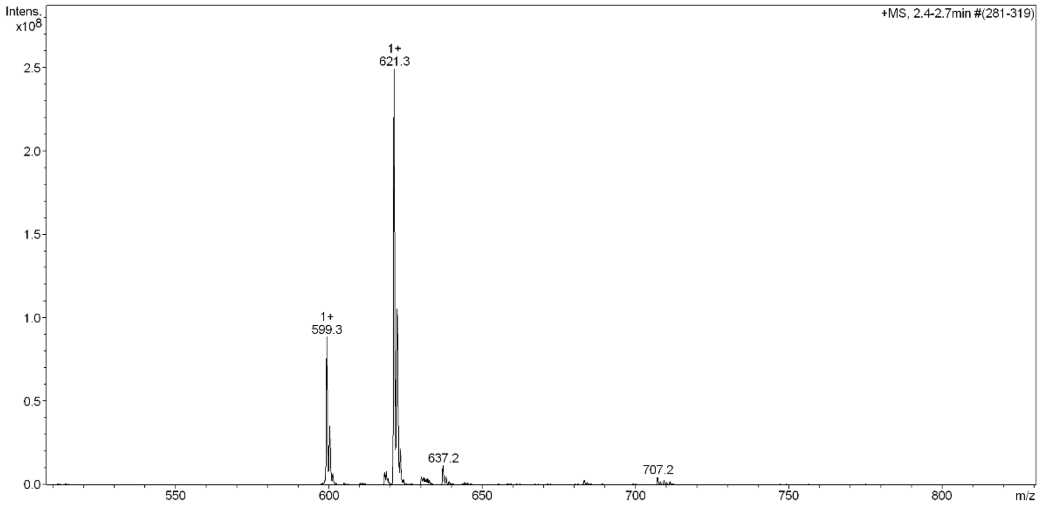

Figure S21. ESI-MS spectrum of **10** (CD_3CN).

[Bis(3,7-dibenzyl-1,5-di(1'-(*R*)-phenylethyl)-1,5-diaza-3,7-diphosphacyclooctane)- tetrafluoroboratoiron(II)] tetrafluoroborate (11b).

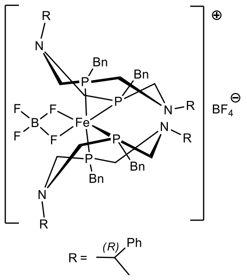

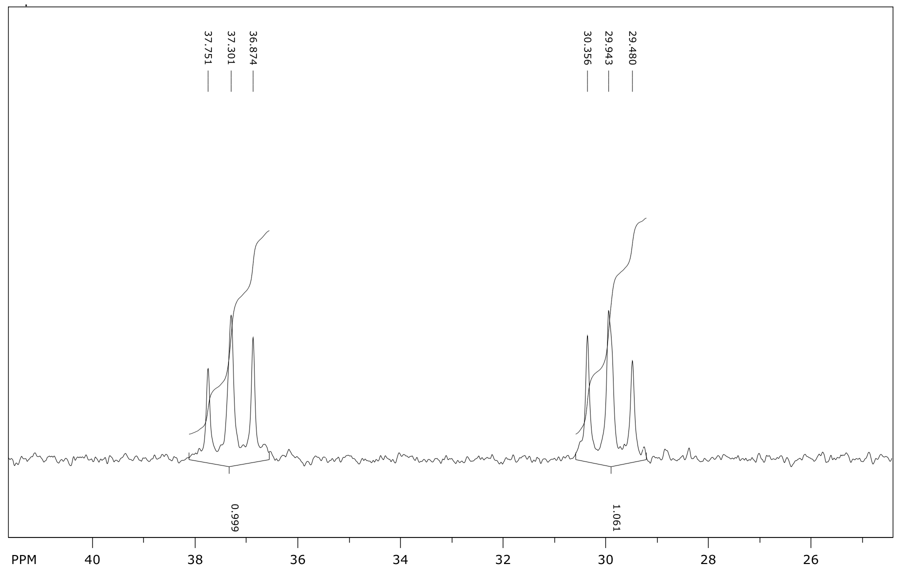

Figure S22. ^{31}P NMR spectrum of **11b** ($(CD_3)_2CO$, 162 MHz).

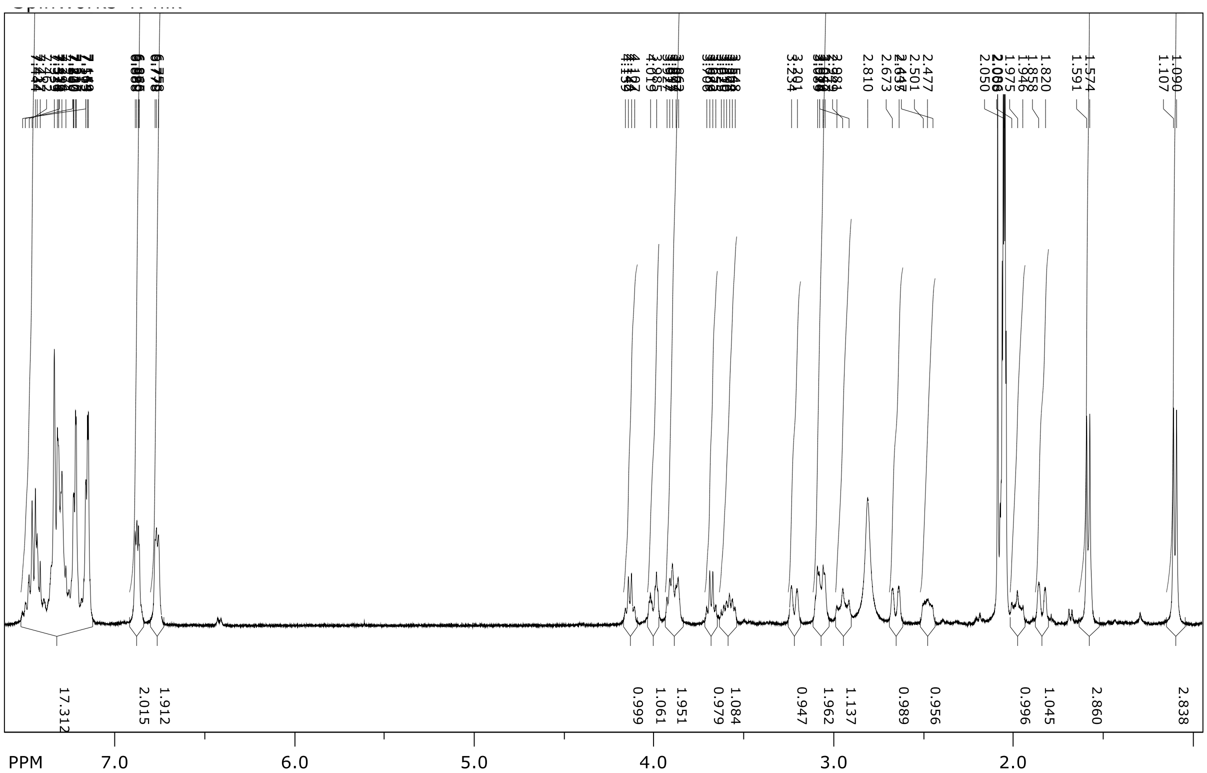

Figure S23. ^{1}H NMR spectrum of **11b** ($(CD_3)_2CO$, 400 MHz) (2.81 s – H_2O in the solvent).

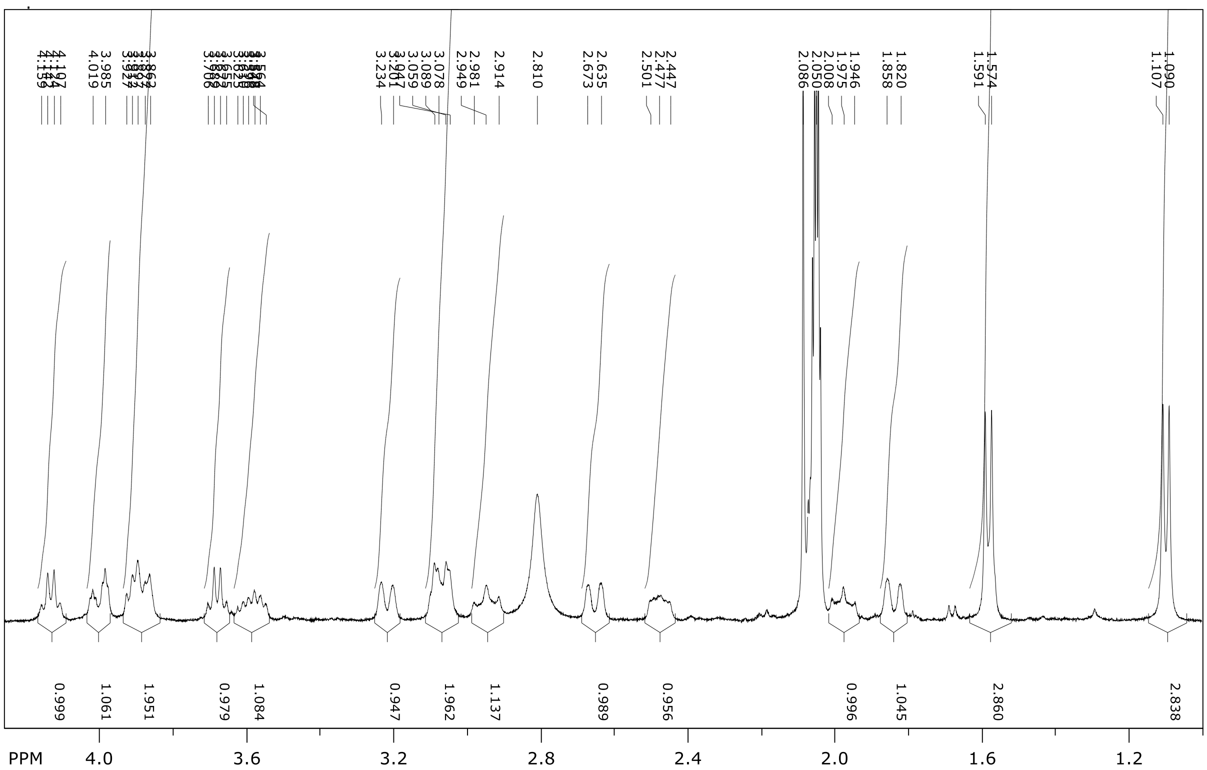

Figure S24. ^{1}H NMR spectrum of **11b** ($(CD_3)_2CO$, 400 MHz), the region of aliphatic protons (2.81 s – H_2O in the solvent).

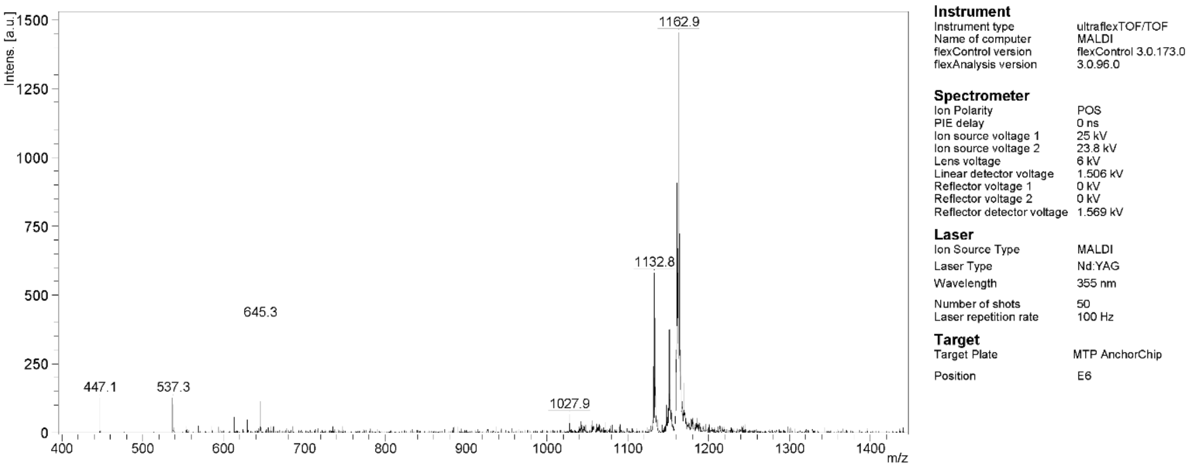

Figure S25. MALDI-MS spectrum of **11b** ($(CH_3)_2CO$).

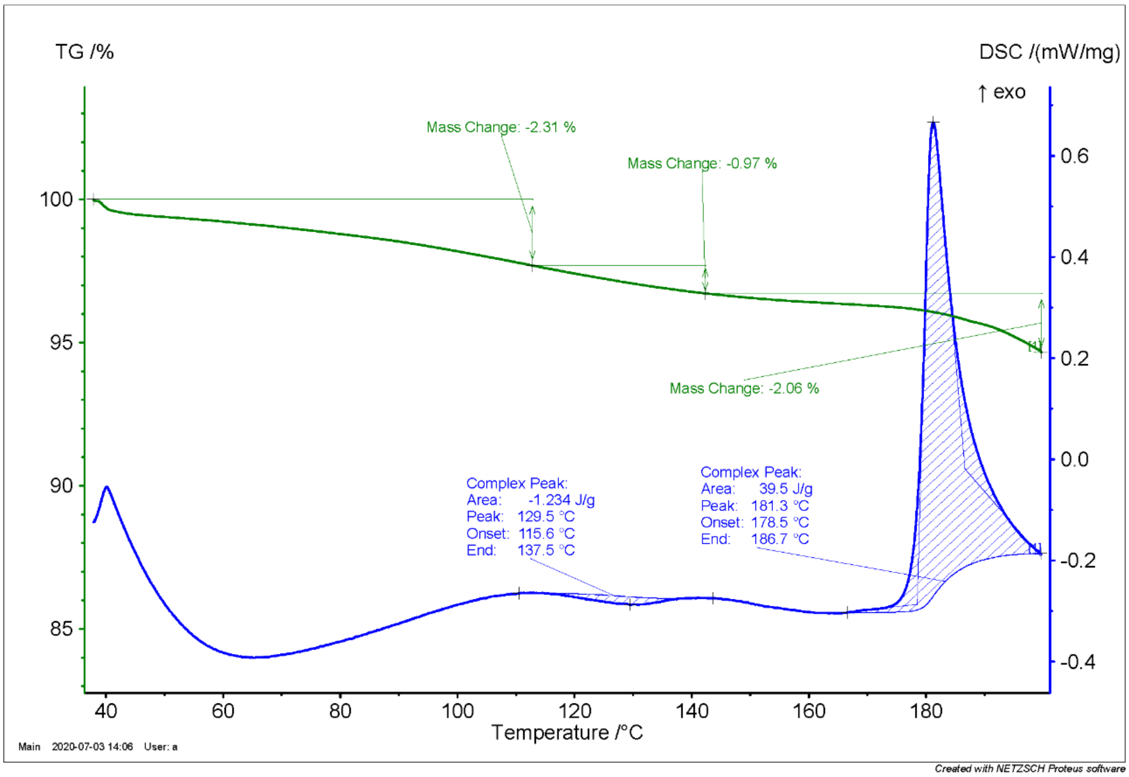

Figure S26. TG/DSK diagram of **11b** (single crystals containing solvate molecules).

[(Cyclopentadienyl)carbonyl(3,7-dibenzyl-1,5-di(1'-(*R*)-phenylethyl)-1,5-diaza-3,7-diphosphacyclooctane)iron(II)] tetrafluoroborate (12).

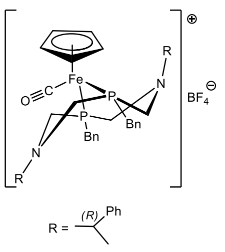

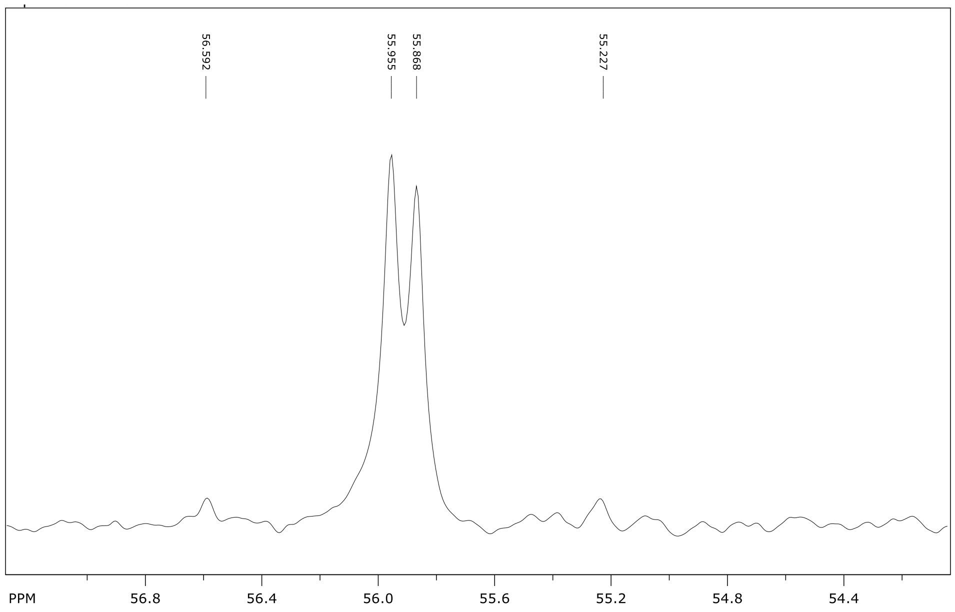

Figure S27. ^{31}P NMR spectrum of **12** ($(CD_3)_2CO$, 162 MHz).

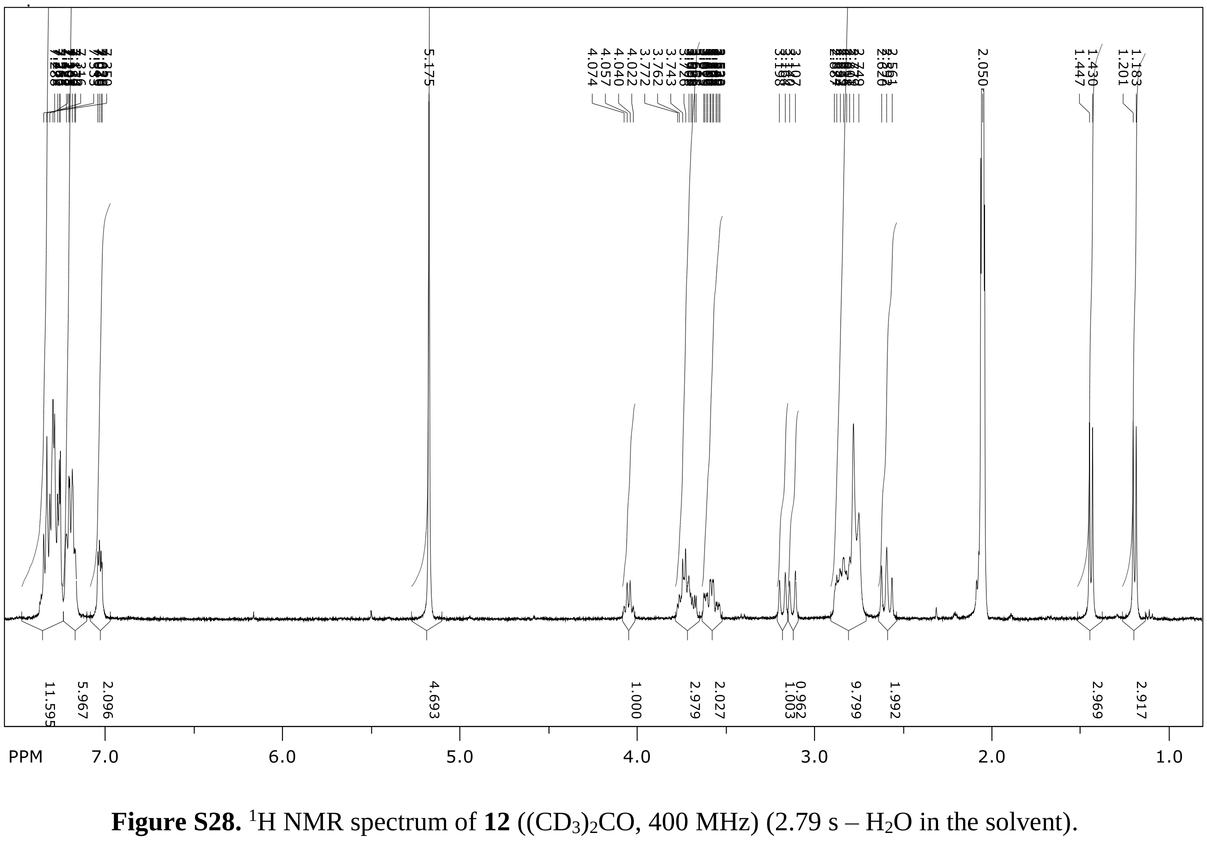

Figure S28. ^{1}H NMR spectrum of **12** ($(CD_3)_2CO$, 400 MHz) (2.79 s – H_2O in the solvent).

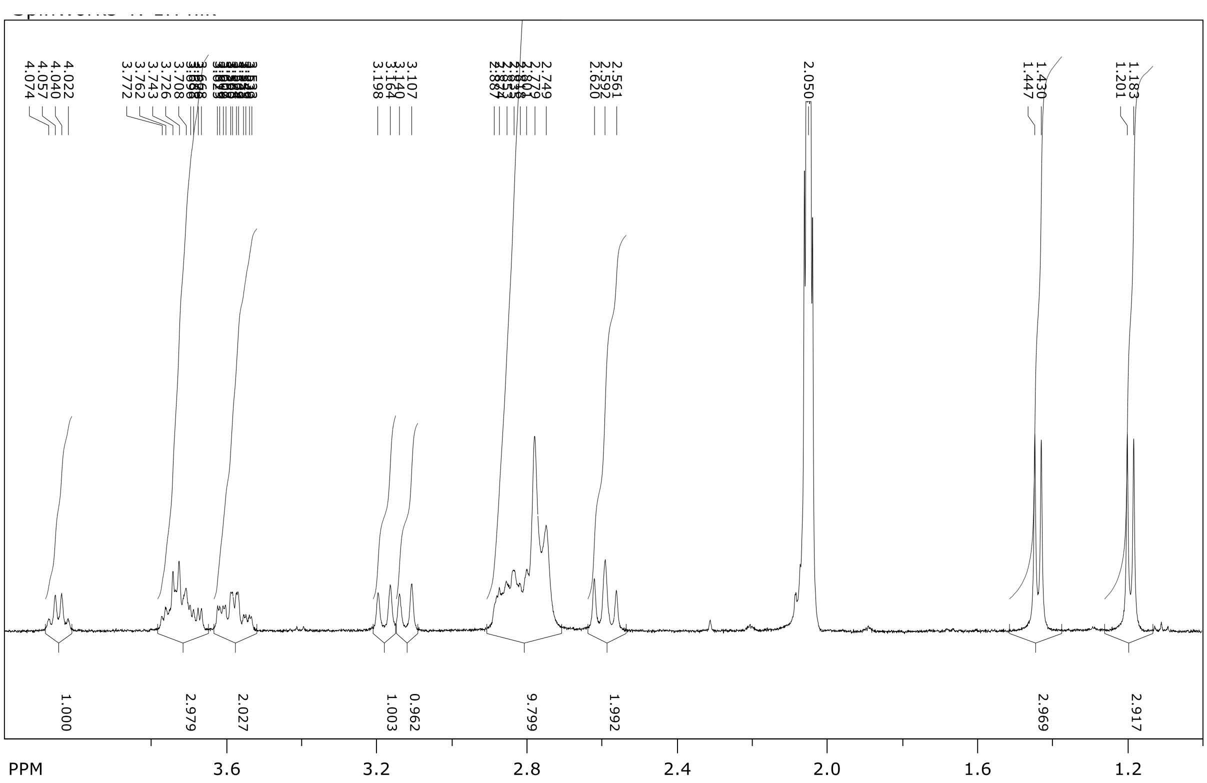

Figure S29. ^1H NMR spectrum of **12**, the region of aliphatic protons ($(CD_3)_2CO$, 400 MHz) (2.79 s – H_2O in the solvent).

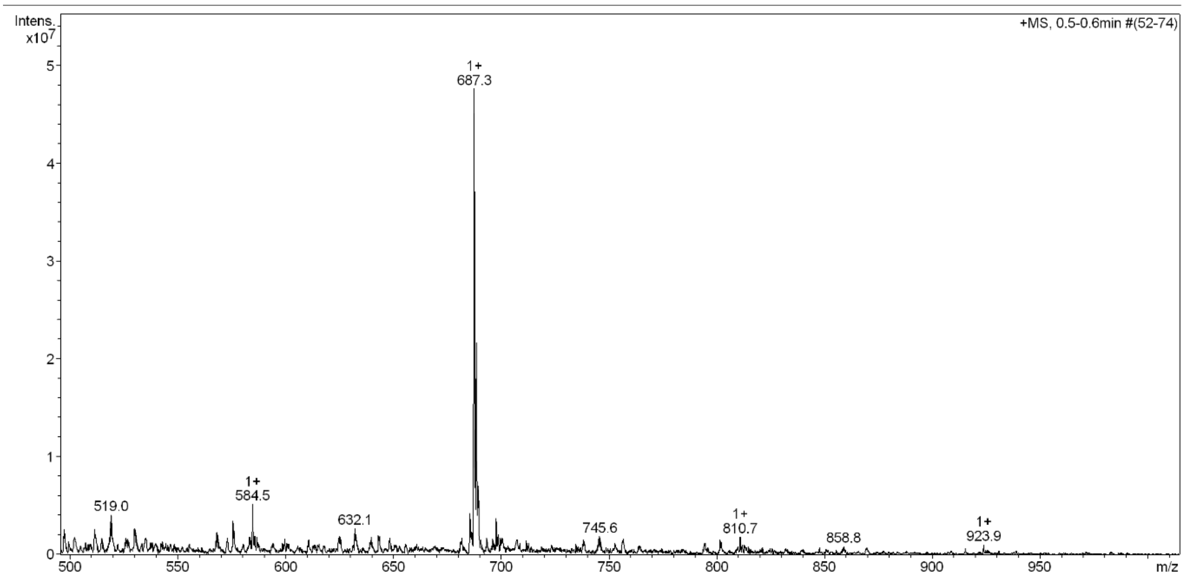

Figure S30. ESI-MS spectrum of **12** ($(CH_3)_2CO$).

[(Cyclopentadienyl)carbonyl(3,7-di(pyridine-2’-yl)-1,5-di(p-tolyl)-1,5-diaza-3,7-diphosphacyclooctane)iron(II)] tetrafluoroborate (13).

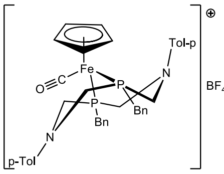

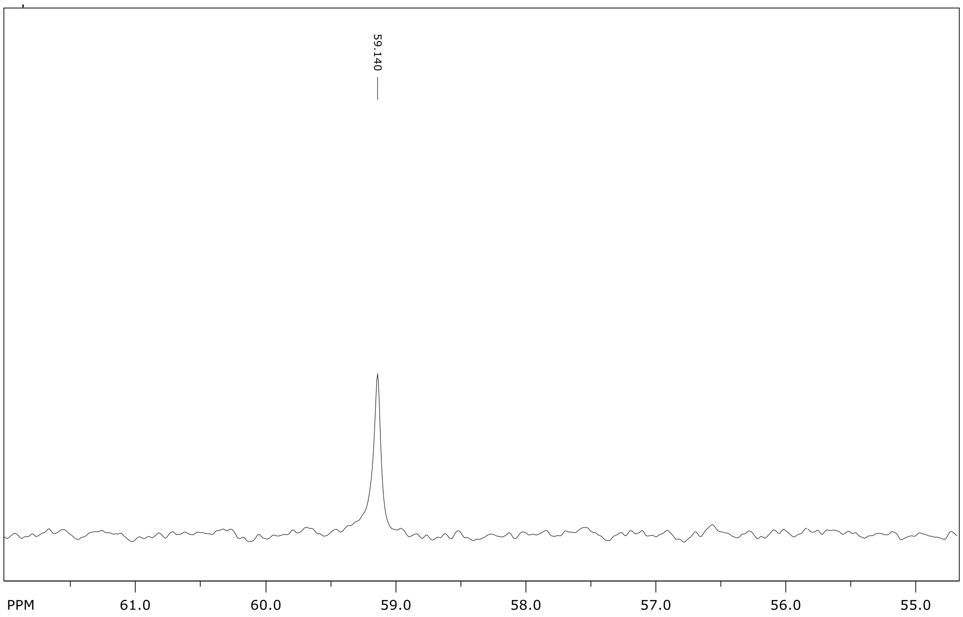

Figure S31. ^{31}P NMR spectrum of **13** ($(CD_3)_2CO$, 162 MHz).

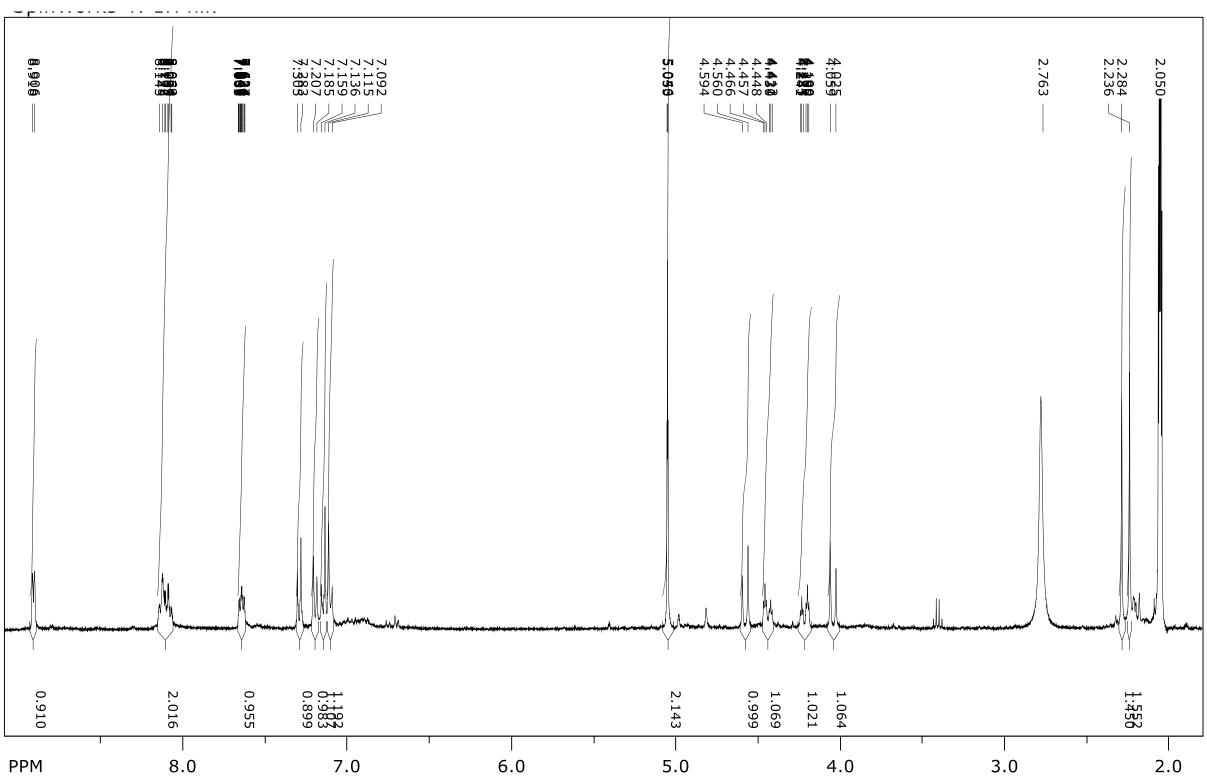

Figure S32. ^{1}H NMR spectrum of **13** ($(CD_3)_2CO$, 400 MHz) (2.76 s – H_2O in the solvent).

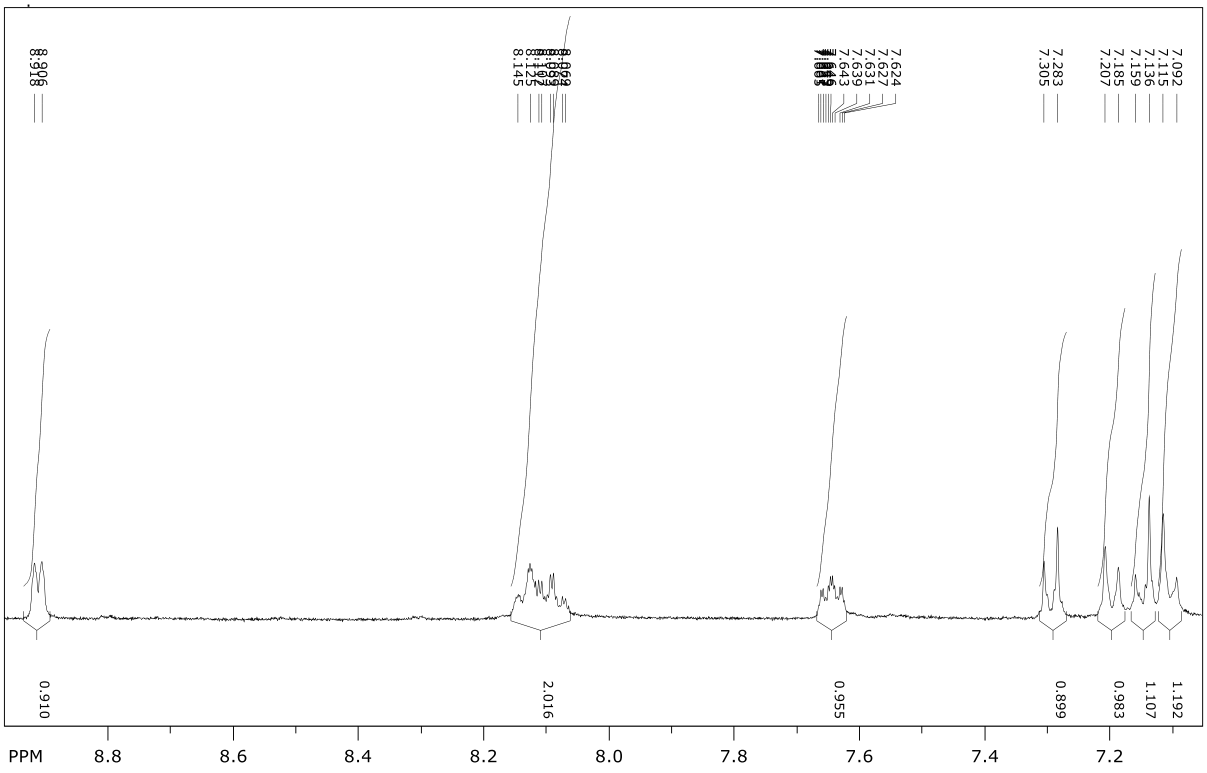

Figure S33. ^{1}H NMR spectrum of **13,** the region of aromatic protons ($(CD_3)_2CO$, 400 MHz)

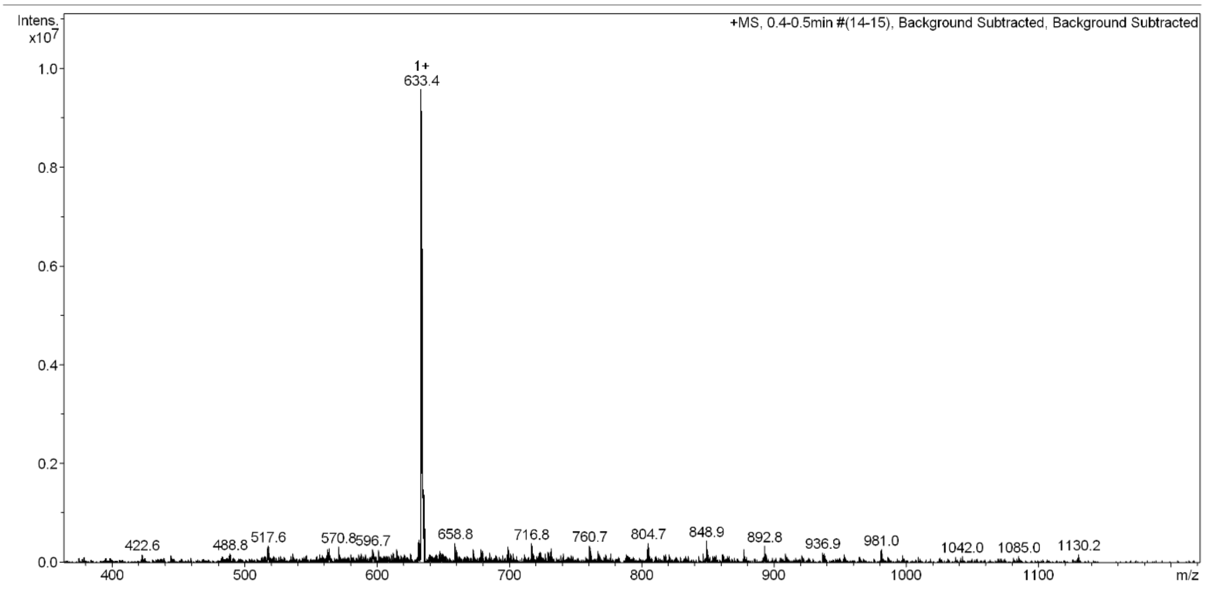

Figure S34. ESI-MS spectrum of **13** ($(CH_3)_2CO$).
